# Integrated Proteomic and Metabolomic Analyses of Cerebrospinal Fluid from Pediatric Patients with Diffuse Intrinsic Pontine Glioma

**DOI:** 10.3390/ijms27156688

**Published:** 2026-07-27

**Authors:** Yufan Chen, Yafei Wang, Yunkun Wang, Kun Zhang, Chenran Zhang

**Affiliations:** 1 Department of Pediatric Neurosurgery, Xinhua Hospital, Shanghai Jiao Tong University School of Medicine, Shanghai 200092, China; yufanchenns@sina.com (Y.C.); wyf0073@sjtu.edu.cn (Y.W.); daqi100@163.com (Y.W.); 2 Shanghai Institute for Pediatric Research, Shanghai Key Laboratory of Pediatric Gastroenterology and Nutrition, Xin Hua Hospital, Shanghai Jiao Tong University School of Medicine, Shanghai 200092, China

**Keywords:** DIPG, CSF, multiomics, immunoglobulins, microbial infections, purine metabolism

## Abstract

Diffuse intrinsic pontine glioma (DIPG) is a rare and fatal pediatric brainstem malignancy for which effective treatment options are lacking. Cerebrospinal fluid (CSF) analysis can reveal intrinsic alterations and characteristic metabolic profiles of the tumor microenvironment. In this study, the proteome and metabolome of CSF from DIPG patients were comprehensively analyzed to identify potential biomarkers and the pathways involved. Functional annotation and pathway enrichment analyses were performed using the GO (Gene Ontology) and KEGG (Kyoto Encyclopedia of Genes and Genomes) databases. Bioinformatics methods were used to comprehensively analyze the proteomic and metabolomic results to identify key differentially expressed proteins, metabolites, and potential signaling pathways involved in DIPG. In total, 885 DEPs (differentially expressed proteins) were identified in cerebrospinal fluid from DIPG patients, of which 54 were upregulated and 831 were downregulated, primarily originating from the cytoplasm and cell membrane. Among the top 20 upregulated proteins, URB1 (nucleolar pre-ribosomal-associated protein 1) had the greatest statistical significance, while the remaining proteins were mostly immunoglobulin fragments. GO enrichment analysis revealed that the downregulated proteins were enriched primarily in cellular processes, metabolic processes, and binding functions. KEGG analysis revealed that upregulated proteins were significantly enriched in complement and coagulation cascades, whereas downregulated proteins were primarily associated with endocytosis and certain microbial infections. A total of 1372 metabolites were identified, of which 40 were differentially expressed: 24 were upregulated, and 16 were downregulated. Pathway analyses of the differentially expressed metabolites revealed that they were primarily related to purine metabolism and tyrosine metabolism. The multiomics analysis revealed that purine metabolism is particularly important in DIPG.

## 1. Introduction

DIPG is a malignant pediatric brainstem tumor that typically arises in the pons. It is closely associated with specific genetic mutations and is a subtype of diffuse midline glioma (DMG) with a very poor prognosis [1]. Despite its low incidence (1.78 cases per 100,000 population), gliomas (including DIPG) remain the leading cause of cancer-related death in children under 19 years of age [2]. DIPG accounts for nearly half of all pediatric high-grade gliomas (HGGs), typically affecting children aged 6 to 8 years. The median survival is less than one year, and the five-year overall survival rate is less than 1% [3,4]. Genetic hallmarks of DIPG include *PDGFRA* activation, *TP53* loss-of-function, and the H3.3 K27M mutation, which are associated with tumor aggressiveness and a poor prognosis. Mutations in genes encoding histone 3 variants (H3.3 or H3.1/H3.2) with a lysine-to-methionine substitution at position 27 can lead to severe dysfunction in epigenetic programming and signaling [5]. Single-cell RNA sequencing (scRNA-seq) studies have shown that DIPG cells are primarily composed of oligodendrocyte precursors (OPCs), which are in a rapidly proliferating state, promoting rapid tumor growth and sustained PDGFRA signaling [6]. Despite advances in understanding DIPG at the molecular level, its prognosis has not improved.

Cerebrospinal fluid is an important source of potential molecular biomarkers. CSF contains a variety of biomarkers, such as circulating tumor DNA (ctDNA), microRNAs, proteins, metabolic molecules, and extracellular vesicles (EVs). Tumors release circulating biomarkers into the blood through the partially damaged blood–brain barrier (BBB), and these biomarkers may also be directly secreted into the CSF [7]. Cerebrospinal fluid is generally considered an extension of the extracellular space of the central nervous system (CNS), and changes in CSF are believed to sensitively reflect CNS pathology and to be an important route for the metastasis of brain tumors [8,9]. In patients with glioblastoma (GBM), a damaged blood–brain barrier allows circulating biomarkers, including certain proteins and metabolites, to enter the blood or CSF. Cerebrospinal fluid is a rich source of tumor-derived cell-free DNA (cfDNA) for the evaluation of central nervous system (CNS) tumors. CSF-based targeted sequencing has the potential to aid brain tumor diagnosis in patients presenting with new intracranial lesions [10]. Recent evidence suggests that cell-free DNA in CSF can serve as a surrogate biomarker of measurable residual disease (MRD) in patients with diffuse midline gliomas, particularly those located in the pons [11].

Because DIPG cannot be surgically removed, tissue samples available for molecular studies are extremely limited, resulting in a poor understanding of its tumor biology. In the absence of frozen tumor samples, body fluids such as cerebrospinal fluid, serum, and urine offer more accessible avenues for detecting tumor-secreted proteins. Currently, only one study has examined the proteome of CSF from DIPG patients [12]. In this study, 76 samples, including CSF, serum, urine, and normal and tumor brainstem tissues, were analyzed, and 528 unique proteins were identified. Cyclophillin A (CypA) and dimethylarginase 1 (DDAH1) were selectively upregulated in the CSF of DIPG patients. This study provides important insights into the tumor biology of DIPG, demonstrating that a CSF analysis can be a noninvasive approach to explore the molecular mechanisms of DIPG. However, this study was published in 2012, when proteomic techniques were not yet fully mature. Given the rapid advances in mass spectrometry and data-independent acquisition (DIA) over the past decade, an updated proteomic profiling of DIPG CSF is warranted.

Metabolic changes in brain tumors disrupt anabolic and catabolic processes, affecting cell signaling pathways and function and thereby promoting tumor development. Circulating metabolites, such as amino acids, carbohydrates, nucleosides, vitamins, and fatty acids, primarily participate in the maintenance of cellular structure and signal transduction through second messenger molecules [13]. Metabolomics can dynamically, comprehensively, and accurately depict disease phenotypes; facilitate the discovery of new biomarkers; and deepen our understanding of disease progression and treatment response, highlighting its importance in the study of disease pathogenesis. Commonly used techniques for CSF metabolomics include magnetic resonance spectroscopy (MRS), a specialized MRI-based technique that detects metabolite concentrations by measuring the chemical shift in proton signals in cerebrospinal fluid, and mass spectrometry, which provides high-sensitivity identification and quantification of metabolites through liquid chromatography–tandem mass spectrometry (LC-MS/MS). MRS does not require sample pretreatment and does not damage or alter the state of metabolites, making it particularly suitable for analyzing trace amounts of CSF. However, due to the low sensitivity of MRI, the tendency for signal overlap, and the difficulty in detecting unconventional metabolites such as lipids and glycoproteins, mass spectrometry is currently more widely used in metabolomic analyses. Metabolomics has great potential in the search for biomarkers for diagnosing diseases. Previous studies have confirmed the metabolomic characteristics of cerebrospinal fluid (CSF) in brain tumors [14]. Using CSF samples from 163 patients with brain diseases, researchers employed high-throughput, nontargeted CSF metabolomics to comprehensively map the entire metabolic profile of primary and secondary central nervous system lymphomas and lung cancer brain metastases. The results of this study revealed that the disrupted metabolic pathways involved primarily amino acid and citric acid metabolism. Currently, no studies have been published on the CSF metabolome of DIPG patients. In our study, we conducted the first comprehensive, integrated proteomic and metabolomic analyses to investigate the multiomics characteristics of CSF from DIPG patients. This study helps identify potential diagnostic and prognostic biomarkers and provides strong evidence for exploring the biological mechanisms of DIPG.

## 2. Results

### 2.1. Proteomic Analysis

**Figure 1:**
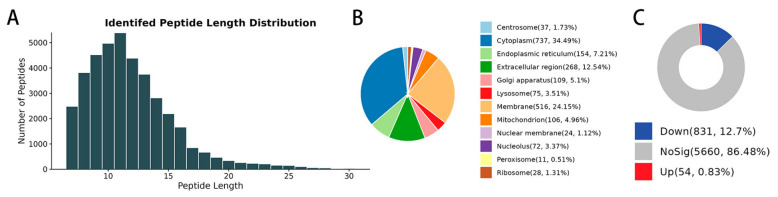
Basic CSF proteomic data: (**A**) This experiment uses a high-quality, high-resolution mass spectrometer to maintain good mass deviation during data acquisition. The threshold for peptide mass error was set to ±10 ppm. The thresholds for the library search and qualitative analysis, a peptide FDR ≤ 0.01 and a protein FDR ≤ 0.01, were used as screening criteria to construct a peptide length distribution map. (**B**) Subcellular localization of the differentially expressed proteins. (**C**) Circular plot of the differentially expressed protein ratios.

**Figure 2:**
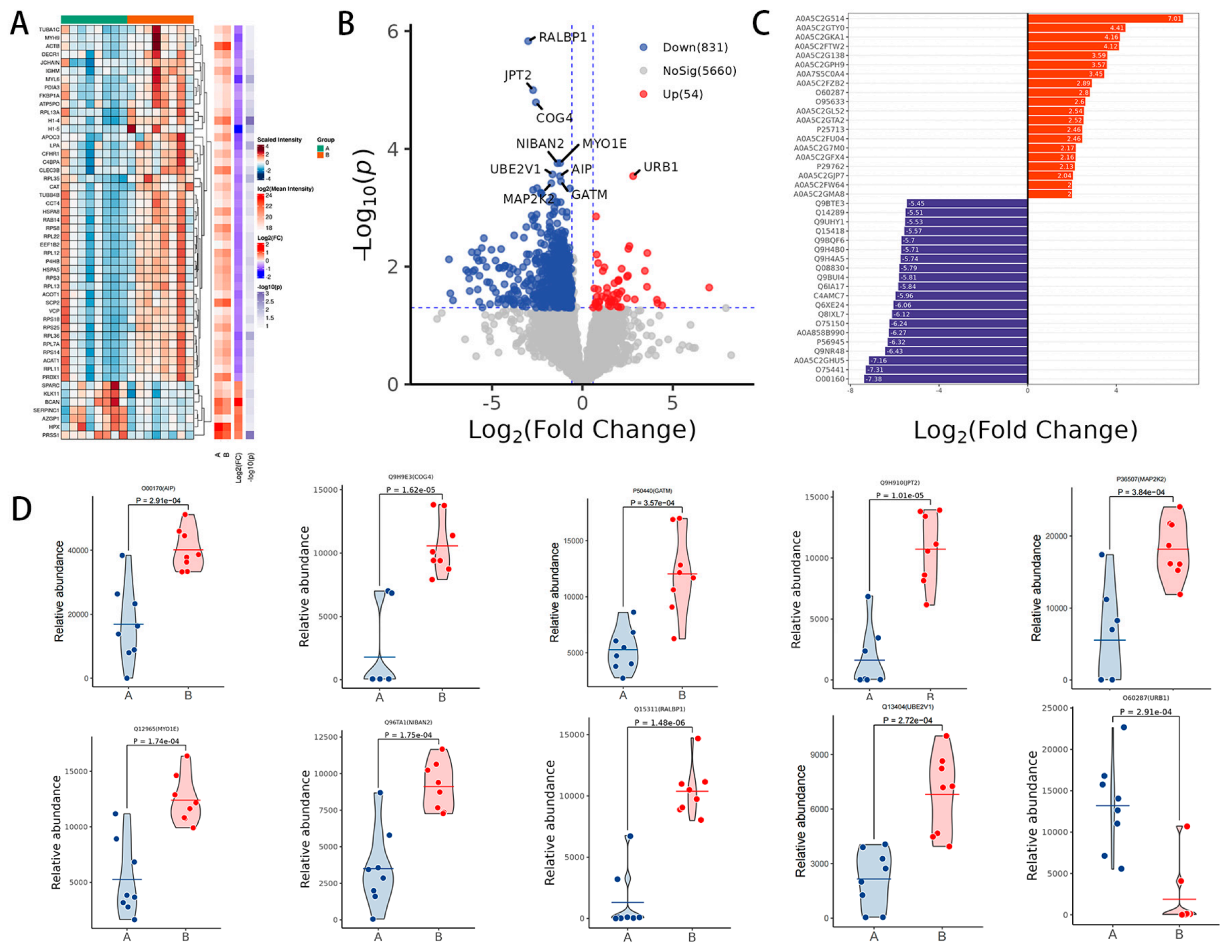
Overview of the differentially expressed proteins in CSF: (**A**) Complex heatmap of the top 50 proteins according to total expression intensity. Columns represent individual samples grouped into Group A (Con) and Group B (DIPG). Rows represent proteins. The color scale on the right represents the signal intensity, log_2_ (Fold Change), and −log_10_ (*p*-value). Red and blue colors indicate up- and downregulation, respectively. (**B**) Volcano plot of differentially expressed proteins between the DIPG and Con groups. The horizontal axis represents the logarithmic transformation of the FC, and the vertical axis represents the negative logarithmic transformation of the *p*-value. Each dot represents a protein, with red dots representing significantly overexpressed proteins, blue dots representing significantly underexpressed proteins, and gray dots representing nondifferentially expressed proteins. (**C**) Bar chart of the fold differences in differentially expressed proteins between the DIPG and Con groups. We plotted the log2 logarithm of the fold difference for the top 20 proteins by both protein accession number and gene name and then plotted the data in a bar chart. The figure below uses the protein accession number as an example. (**D**) The final selected dysregulated proteins.

**Figure 3:**
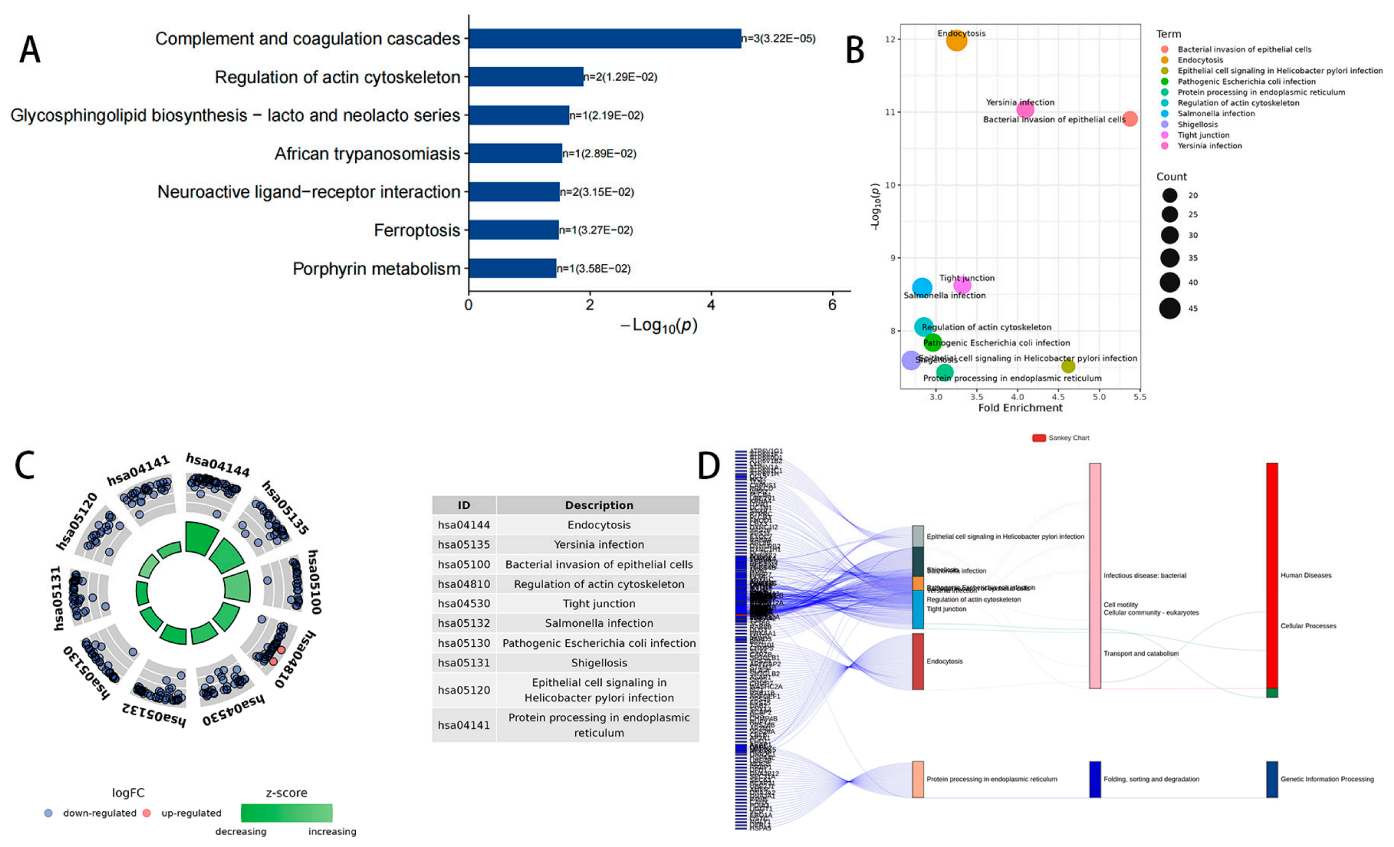
KEGG enrichment analysis of the differentially expressed proteins: (**A**) KEGG pathways significantly enriched in upregulated proteins. The horizontal axis represents the negative logarithmic transformation of the *p*-value, and the vertical axis represents the pathway. The figure also shows the ratio of upregulated to downregulated proteins within the same pathway, represented by different colors. The specific count (number of differentially enriched proteins in that pathway) and *p*-value are indicated to the right of each column. (**B**) The top 10 pathways significantly enriched in downregulated proteins are shown in a bubble chart, which simultaneously displays the *p*-value, fold enrichment, and count parameters in a single graph. The horizontal axis represents the fold enrichment, the vertical axis represents the negative logarithmic transformation of the *p*-value, the circle size represents the count, and the different colors represent individual pathways. (**C**) The figure below shows the top 10 KEGG pathways for all the differentially expressed proteins by *p*-value. The left figure shows, from the outer circle inward, the function number, the protein corresponding to the function (one dot represents one protein; blue indicates downregulation; red indicates upregulation; the *Y*-axis coordinate of the fan is log2(FC); and the *X*-axis coordinate is randomly generated), and the Z-score value of the function. The top ten pathways by *p*-value are labeled with the pathway number in the bubble plot and listed in the table on the right. (**D**) Sankey diagram of the relationships between differentially expressed proteins and pathways; from left to right, the differentially expressed proteins (upregulated in red and downregulated in blue), pathways, pathways in the second-level category, and pathways in the top-level category.

This study included a cohort of eight patients with DIPG and another eight patients diagnosed with arachnoid cysts as a control group. Patients and controls were matched for age and sex. Quantitative proteomics was performed using DIA (data-independent acquisition) with a high-quality, high-resolution mass spectrometer to maintain good mass deviation during data acquisition. The peptide mass error threshold was set at ±10 ppm. A peptide FDR ≤ 0.01 and a protein FDR ≤ 0.01 were used as screening criteria for library identification, along with the peptide score distribution. The peptide length distribution (Figure 1A) demonstrated high data quality for subsequent analysis and reliable identification results. A total of 6545 proteins were identified and quantified. The subcellular localization analysis revealed that the top three subcellular locations were the cytoplasm (34.49%), cell membrane (24.15%), and extracellular space (12.5%) (Figure 1B). A total of 885 proteins were identified as differentially expressed proteins (DEPs), and the vast majority (approximately 94%) were downregulated in the DIPG group compared with the normal control group. Of these, 54 proteins were upregulated, and 831 were downregulated (Figure 1C). We selected the top 50 proteins by overall expression level and plotted them in a complex heatmap (Figure 2A). The DEPs are visualized in the volcano plot shown in Figure 2B. Among the upregulated DEPs, URB1 had the greatest statistical significance. Among the downregulated DEPs, the five most significantly downregulated proteins were RALBP1 (ralA-binding protein 1), JPT2 (jupiter microtubule-associated homolog 2), COG4 (conserved oligomeric golgi complex subunit 4), NIBAN2 (protein niban 2), and MYO1E (unconventional myosin-Ie). We selected the top 20 proteins based on fold change in both the protein accession number and gene name and plotted the fold changes using the log2 logarithm (Figure 2C). The majority of the upregulated proteins were immunoglobulin fragments, and others, including URB1, FSTL3, MT3, and CRABP1, were upregulated. The downregulated proteins included MYO1F, ASH1L, BCAR1, HLA-A, RNF40, MSRB3, RBMS3, WASH3P, SIGIRR, POLR3C, FGL1, GOLPH3L, OSGEPL1, SENP7, RPS6KA1, NRBP1, PTK2B, and MCMBP (Table 1 and Table 2). Violin plots of the expression of the most significantly dysregulated proteins among these differentially expressed proteins are shown in Figure 2D. After the DEPs were identified, GO/KEGG enrichment analyses were performed to characterize their functions. All the DEPs were divided into three possible categories: biological processes (BPs), cellular components (CCs), and molecular functions (MFs). As shown in Supplementary Figure S1A, the top five BPs for the downregulated proteins were cellular process, metabolic process, biological regulation, regulation of biological process, and response to stimulus. The top two CCs were cellular anatomical entities and protein-containing complexes. The top two MF terms were primarily associated with binding and catalytic activity. As shown in Supplementary Figure S1B, for the upregulated proteins, the top five BPs were identical to those for the downregulated proteins: cellular process, metabolic process, biological regulation, regulation of biological process, and response to stimulus. The top two CC terms were also the same as those for the upregulated proteins. The top three MF terms were primarily associated with binding, molecular function regulator activity, and catalytic activity. A GO analysis of all the genes in the species is shown in Supplementary Figure S2. The KEGG pathway analysis revealed that the upregulated proteins were significantly enriched in complement and coagulation cascades (Figure 3A). The downregulated proteins were enriched primarily in endocytosis, Yersinia infection, bacterial invasion of epithelial cells, tight junctions, Salmonella infection, regulation of actin cytoskeleton, pathogenic Escherichia coli infection, shigellosis, epithelial cell signaling in Helicobacter pylori infection, and protein processing in endoplasmic reticulum pathways (Figure 3B). For all DEPs, the enriched pathways were the same as those for the downregulated proteins (Figure 3C). The Sankey diagram of the relationships between the differentially expressed proteins in the top 10 pathways and the pathways is shown in Figure 3D.

### 2.2. Metabolomic Analysis

**Figure 4:**
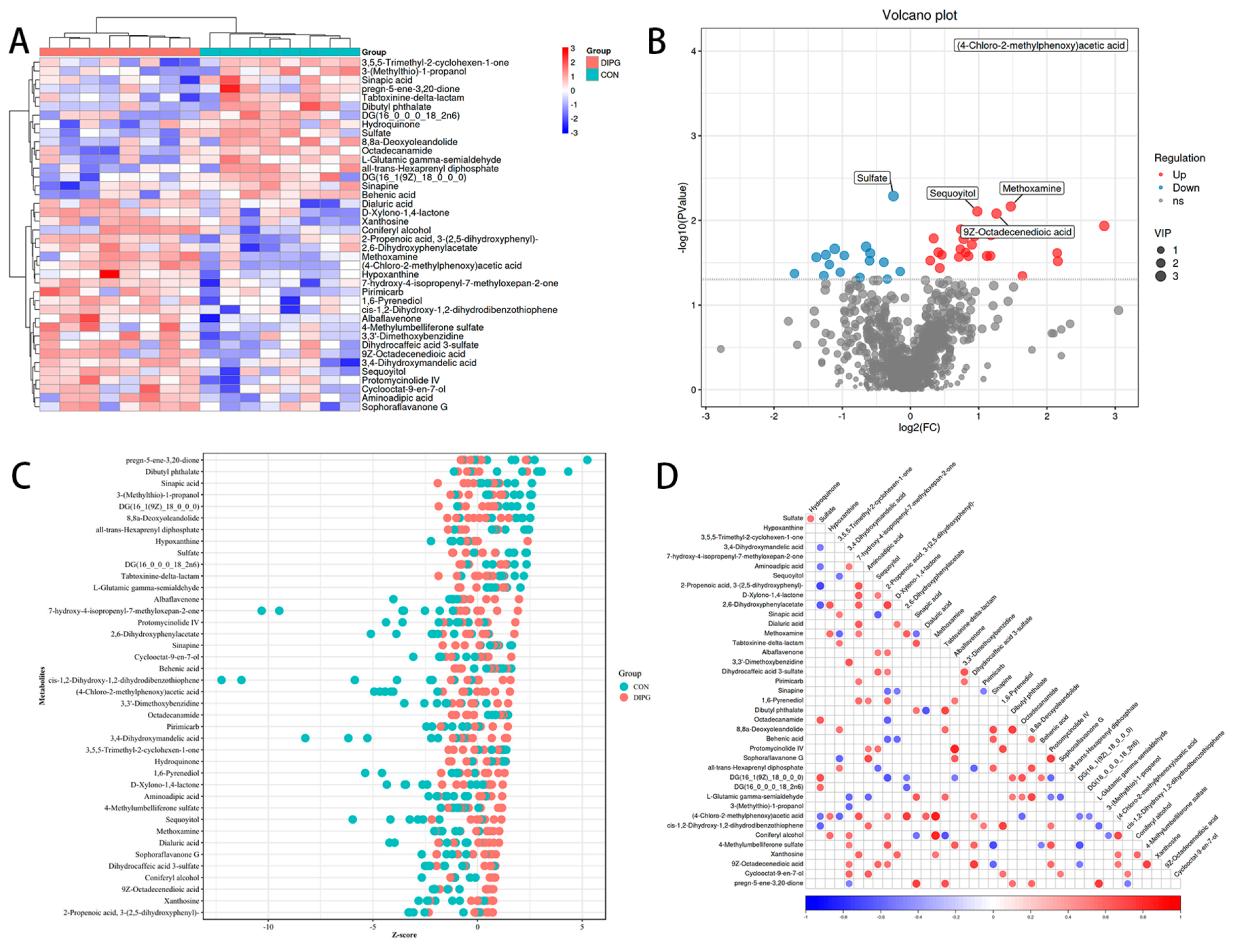
Overview of the secondary metabolite profiles: (**A**) Differentially abundant metabolite cluster heatmap. Columns represent samples, and rows represent metabolites. The cluster tree on the left is the differentially abundant metabolite cluster tree, and the top is the sample cluster tree. The gradient color represents the magnitude of the quantitative value: redder colors indicate higher expression levels, and bluer colors indicate lower expression levels. Metabolite names are not displayed for metabolites with more than 150 metabolites. (**B**) Volcano plot of differentially abundant metabolites. The horizontal axis represents the log2 fold difference in the quantitative value of a metabolite between the two samples; the vertical axis represents the -log_10_ *p*-value. Each dot in the plot represents a metabolite. A larger absolute value on the horizontal axis indicates a greater fold difference in expression between the two samples; a larger value on the vertical axis indicates more significant differential expression and a more reliable differentially expressed metabolite. The size of the dot represents the VIP value. Red dots represent differential upregulation, blue dots represent differential downregulation, and gray dots indicate metabolites that do not meet the differential screening criteria. The names of the five metabolites with the lowest *p*-values are displayed by default. (**C**) Z-score plot of secondary differentially abundant metabolites. The horizontal axis represents the Z-score value of the metabolite’s relative abundance in the sample, and the vertical axis represents the metabolite’s name. The color of the dots represents the different groups. The closer to the right, the higher the relative abundance of the metabolite in the sample; the closer to the left, the lower the relative abundance. (**D**) Correlation heatmap of secondary differentially abundant metabolites. Both the vertical and oblique vertical axes represent the names of the differentially abundant metabolites. The color represents the correlation, with red indicating a positive correlation and blue indicating a negative correlation. Darker colors indicate stronger correlations.

**Figure 5:**
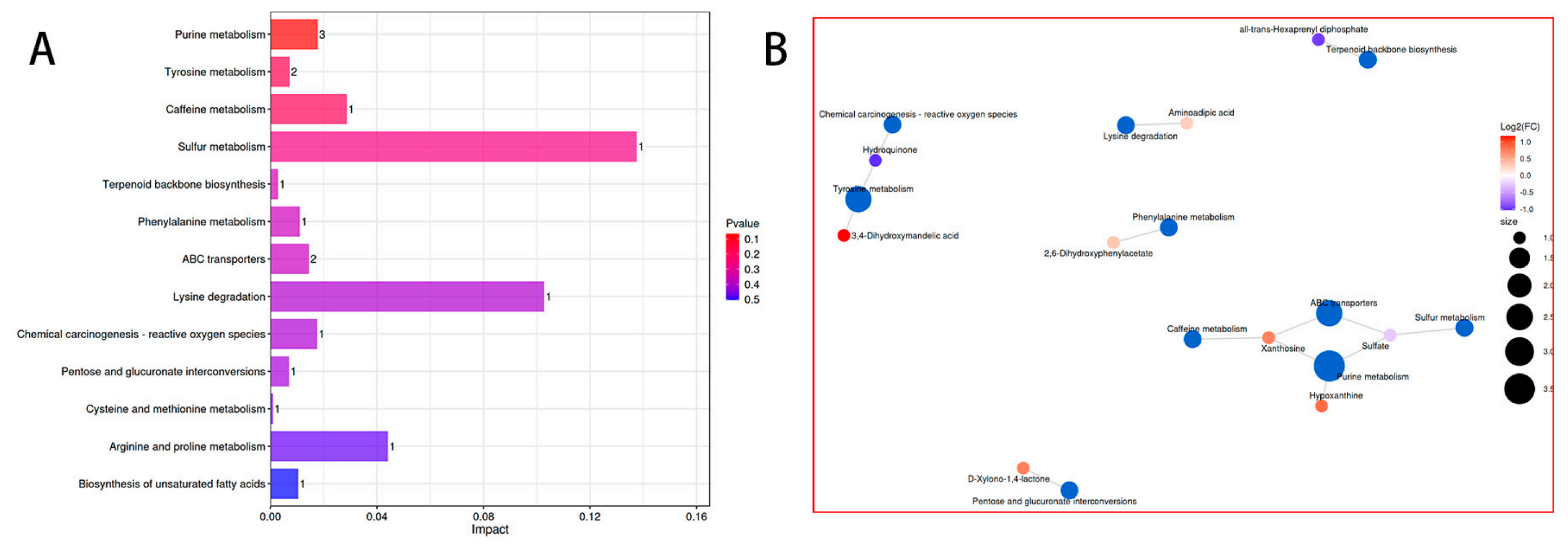
KEGG pathway enrichment analysis of the differentially abundant metabolites: (**A**) Bar chart of the metabolic pathway impact factors. The horizontal axis represents the impact value of enrichment in different metabolic pathways, and the vertical axis represents the metabolic pathways. The numbers indicate the number of metabolites corresponding to the pathway. The color is correlated with the *p*-value: the redder the color, the smaller the *p*-value, and the bluer the color, the larger the *p*-value. (**B**) The blue dots represent pathways, and the other dots represent metabolites. The size of the dot for each pathway represents the number of metabolites connected to it; the larger the number of connections, the larger the dot. The color of the dots for the metabolites represents the size of the log2(FC) value: red indicates differential upregulation, blue indicates differential downregulation, and darker colors indicate greater differences.

Differentially abundant metabolites were identified from the primary metabolite list and analyzed using predefined statistical tests. Significant differences were screened using *p*-values and VIP thresholds. Significant changes were detected in DIPG patients in positive and negative ion modes for 7835 metabolites (118 upregulated and 77 downregulated) (Supplementary Figure S3) and 5985 metabolites (89 upregulated and 89 downregulated), respectively (Supplementary Figure S4). Substances were identified using spectral databases such as HMDB, MassBank, LipidMaps, mzcloud, KEGG, and a self-built metabolite standard database. Metabolites with secondary spectra in the quantification list were compared and matched with fragment ions and other information from each secondary spectrum in the database to achieve secondary qualitative identification. Secondary metabolites were selected from the identification results and screened using the *p*-value and VIP thresholds predefined in the primary differential expression analysis results to identify secondary differentially abundant metabolites. A total of 1372 metabolites were identified, including 40 differentially abundant metabolites, 24 of which were upregulated and 16 of which were downregulated (Figure 4). The 24 upregulated metabolites are listed in Table 3. The 16 downregulated metabolites are listed in Table 4. The pathway analysis revealed that the differentially abundant metabolites were primarily related to purine metabolism and tyrosine metabolism (Figure 5, Table 5).

### 2.3. Integrated Bioinformatics Analysis of Proteomics and Metabolomics

**Figure 6:**
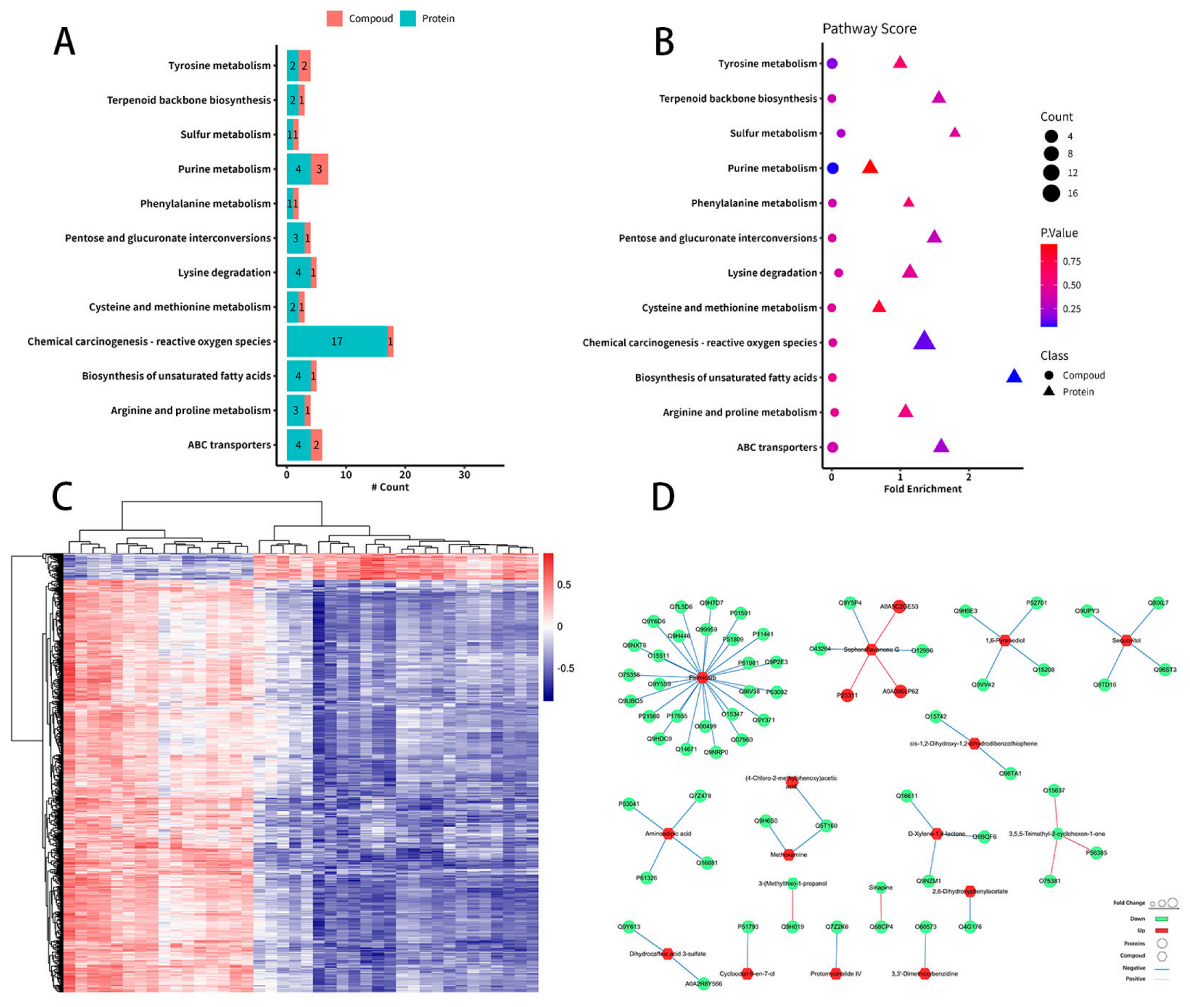
Correlation analysis of the proteomic and metabolomic data: (**A**) KEGG pathway mapping analysis. We performed a KEGG pathway annotation analysis for both differentially expressed proteins and differentially expressed metabolites to investigate potential pathway connections between differentially expressed proteins and differentially expressed metabolites. We then screened for pathways containing both differentially expressed proteins and differentially expressed metabolites. (**B**) KEGG pathway enrichment analysis. Fisher’s exact test was used to perform the KEGG pathway enrichment analysis of differentially expressed proteins and metabolites. Pathways were considered significantly enriched when the *p*-value was <0.05. (**C**) Spearman’s correlation analysis. Proteomic and metabolomic association analyses were performed on differentially expressed proteins and differentially metabolized small molecules, and Spearman’s correlation coefficients were calculated between each pair. The horizontal axis represents metabolite clustering, whereas the vertical axis represents protein clustering. Shorter cluster branches indicate greater similarity, and redder colors indicate stronger positive correlations. Bluer colors indicate stronger negative correlations. (**D**) Coexpression network of differentially expressed metabolites and differentially expressed proteins. Using the Spearman correlation analysis above, we screened for expression networks with Spearman’s correlation coefficients greater than 0.6 between metabolites and proteins (these values were dynamically adjusted based on the data in the coexpression network to optimize visualization). This network was revisualized using Cytoscape, resulting in the following network diagram.

We simultaneously constructed regulatory networks for the differentially expressed metabolites and proteins using the Metscape plugin in Cytoscape (version 3.10.2). This network, which is based on existing knowledge from databases, experiments, and the literature, tracks connections between metabolites and genes, visualizes compound networks, and displays compound structures, reaction types, enzymes, genes, and pathway information. This network also allows the validation of differentially expressed proteins at a metabolic level closer to the phenotype (Supplementary Figure S5). We annotated KEGG pathways for both differentially expressed proteins and differentially expressed metabolites to investigate potential pathways connecting the differentially expressed proteins and metabolites. Pathways in which both differentially expressed proteins and metabolites were present were identified, and the statistical results are shown in Figure 6A. The pathways included tyrosine metabolism, terpenoid backbone biosynthesis, sulfur metabolism, purine metabolism, phenylalanine metabolism, pentose and glucuronate interconversions, lysine degradation, cysteine and methionine metabolism, chemical carcinogenesis reactive oxygen species, biosynthesis of unsaturated fatty acids, arginine and proline metabolism, and ABC transporters. Among them, purine metabolism was enriched in most proteins and metabolites (four proteins and three metabolites); thus, we focused on this metabolic pathway. We used Fisher’s exact test to perform a KEGG pathway enrichment analysis on the differentially expressed proteins and metabolites. A *p*-value < 0.05 indicated a significantly enriched pathway (Figure 6B). For the proteomic–metabolomic association analysis, Spearman’s correlation coefficients were calculated for each differentially expressed protein and metabolite pair (Figure 6C). Using the Spearman correlation analysis above, we identified expression networks with Spearman’s correlation coefficients between metabolites and proteins greater than 0.6 (this value was dynamically adjusted based on data from the coexpression network to optimize visualization). This network was revisualized using Cytoscape, resulting in the network diagram shown in Figure 6D.

**Figure 7:**
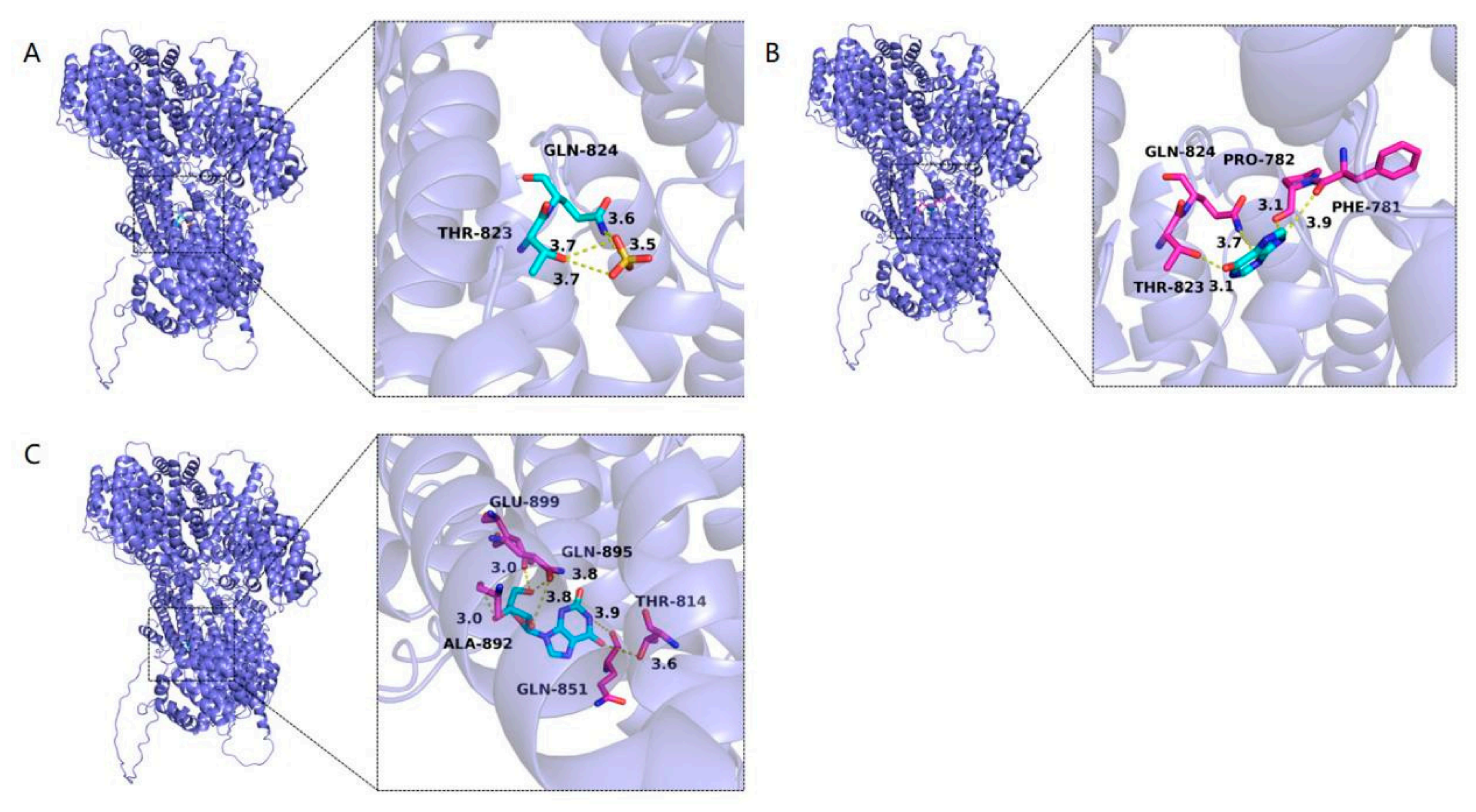
Molecular docking analysis of URB1 interactions with sulfate, hypoxanthine, and xanthosine: (**A**) URB1 and sulfate. The protein is represented as a slate cartoon model, the ligand is shown as a cyan stick, and their binding sites are shown as magenta stick structures. Nonpolar hydrogen atoms are omitted. The hydrogen bond, ionic interactions, and hydrophobic interactions are depicted as yellow, magenta, and green dashed lines, respectively. There are multiple groups of residues used to form interactions between the receptor protein and the ligand, such as the hydrogen bond formed by GLN824 of URB1 and the ligand. With these interaction forces, the binding energy of the protein–ligand complex was −2.9 kcal/mol, which is a poor performance. (**B**) URB1 and hypoxanthine. The hydrogen bond formed by PRO782 of URB1 and the ligand. With these interaction forces, the binding energy of the protein–ligand complex was −5.0 kcal/mol, which is a good performance. (**C**) URB1 and xanthosine. There are multiple groups of residues used to form interactions between the receptor protein and the ligand, such as the hydrogen bond formed by GLU899 of URB1 and the ligand. With these interaction forces, the binding energy of the protein–ligand complex was −5.9 kcal/mol, which is a good performance.

The docking scores with sulfate, hypoxanthine, and xanthosine were −2.9, −5.0, and −5.9 kcal/mol, respectively. URB1 formed multiple interactions with hypoxanthine (a hydrogen bond with PRO782) and xanthosine (a hydrogen bond with GLU899), while only a single hydrogen bond with GLN824 was observed for sulfate. The relatively weak binding of sulfate contrasted with the moderate-to-good binding of the two purine derivatives (Figure 7). For RALBP1, the binding energies were −2.6 kcal/mol (sulfate), −4.4 kcal/mol (hypoxanthine), and −5.4 kcal/mol (xanthosine). RALBP1 established multiple contacts with hypoxanthine (ASP218) and xanthosine (ARG381), whereas sulfate engaged in only one hydrogen bond with LEU255. Again, hypoxanthine and xanthosine showed superior binding affinity (Supplementary Figure S6). For JPT2, the docking energies were −2.0 kcal/mol (sulfate), −4.1 kcal/mol (hypoxanthine), and −4.7 kcal/mol (xanthosine). JPT2 formed multiple hydrogen bonds with hypoxanthine (ALA44) and xanthosine (ASN46) but only a single interaction with sulfate (ASN41). Collectively, among the three metabolites, xanthosine consistently exhibited the strongest binding to all three proteins, followed by hypoxanthine, while sulfate displayed negligible affinity. These results suggest that hypoxanthine and xanthosine may have functional relevance in modulating the activity of the identified proteins (Supplementary Figure S7).

## 3. Discussion

Cerebrospinal fluid (CSF) contains a variety of biomolecules secreted by normal brain tissue or tumor cells, and accumulating evidence suggests that CSF proteomics can reveal potential biomarkers for central nervous system tumors [15,16,17,18]. Notably, CSF immune cytokines have been shown to predict intracranial tumor responses to immunotherapy, and systematic proteomic approaches have provided insights into the tumor microenvironment of leptomeningeal metastases [19,20]. In summary, proteomic research on cerebrospinal fluid from patients with glioma can provide better strategies for glioma diagnosis, grading, and the identification of recurrence and metastasis. However, for pediatric DIPG, CSF-based proteomic and metabolomic studies remain extremely limited. In this study, we performed comprehensive proteomic and metabolomic analyses of CSF from DIPG patients to identify key differentially expressed proteins, metabolites, and the signaling pathways involved.

The molecular biology of brainstem gliomas in pediatric patients differs from that of adults and pediatric patients with supratentorial gliomas, potentially resulting in unique biological characteristics. Given the scarcity of DIPG tissue samples for analysis, innovative research approaches are urgently needed to elucidate the pathogenesis and therapeutic targets of DIPG. Currently, only one study has examined changes in protein expression in the cerebrospinal fluid of DIPG patients, identifying 109 upregulated proteins whose functions are related to tumorigenesis, including 71 involved in the p53 pathway and 68 involved in the AKT pathway. The authors also demonstrated the selective upregulation of extracellular secretory, but not cytoplasmic, CypA and DDAH1, identifying cyclophilin A (CypA) as a monitoring biomarker and potential therapeutic target for pediatric diffuse intrinsic pontine gliomas. They also proposed dimethylarginine dimethylamine hydrolase 1 (DDAH1) as a prognostic factor and indicator of glioma aggressiveness [12]. Our study identified and quantified a relatively large number of proteins, totaling 6545. These proteins were primarily localized in the cytoplasm and cell membrane. A total of 885 proteins were identified as differentially expressed proteins (DEPs), which significantly exceeded the number of proteins identified in previous studies. Interestingly, the vast majority of DEPs were downregulated, suggesting that suppressed cellular biological functions are closely linked to tumorigenesis in DIPG. Among the upregulated DEPs, URB1 had the greatest statistical significance. Among the downregulated DEPs, the five most significant proteins were RALBP1 (RalA-binding protein 1), JPT2 (Jupiter microtubule-associated homolog 2), COG4 (conserved oligomeric Golgi complex subunit 4), NIBAN2 (protein Niban 2), and MYO1E (unconventional myosin-1e). RalA (Ras-like A) is a key downstream effector of Ras. RalA-binding protein 1 (RALBP1) is a key effector of RalA. RALBP1-related EPS domain-containing protein 2 (REPS2) was discovered in the 1990s and is involved in the endocytosis of growth factor receptors [21]. Researchers have observed that REPS2 is involved in the inhibitory effects of the LXR agonist T317 on the proliferation and migration of liver cancer cells. Furthermore, the authors found that T317 promoted REPS2 expression in HepG2 cells, thereby inhibiting epidermal growth factor-mediated EGFR endocytosis and the activation of downstream signaling pathways such as AKT/NF-κB, p38MAPK, and ERK1/2, providing new insights into the treatment of liver cancer [22]. Similar studies have shown that RALBP1 is closely associated with the occurrence and prognosis of prostate cancer, breast cancer, esophageal squamous cell carcinoma, colorectal cancer, and melanoma [23,24,25]. Studies have also shown that RalA is overactivated in medulloblastoma [26]. Currently, no studies have investigated the role of RALBP1 in brainstem gliomas. Jupiter microtubule-associated homolog 2 (JPT2) has been identified as a nicotinic acid adenine dinucleotide phosphate (NAADP)-binding protein and is a key molecule in NAADP-mediated calcium mobilization. Furthermore, JPT2 is a newly discovered oncogene in cancer and has been reported to promote the proliferation and metastasis of various cancer cells [27]. Currently, no clear studies have directly linked JPT2 to gliomas.

Under normal conditions, immunoglobulin (Ig) levels in CSF are extremely low, as the blood–brain barrier (BBB) restricts the entry of large molecules. The marked elevation of immunoglobulin fragments—16 of the top 20 upregulated proteins in our study—raises the possibility of either local intrathecal immune activation or increased BBB permeability in DIPG patients. However, we acknowledge that direct evidence of BBB damage (CSF/serum albumin ratio) was not available in our dataset; therefore, this interpretation remains speculative and warrants further investigation.

We performed GO and KEGG analyses of the differentially expressed proteins. The analyses revealed that the downregulated proteins were enriched primarily in cellular and metabolic processes. Cellular processes encompass a wide range of processes occurring within cells, including cellular metabolism, cell signaling, cell cycle regulation, cell differentiation, apoptosis, and many other specific processes related to cellular function. Further analysis revealed that the downregulated proteins were primarily related to the localization and transport of biomacromolecules (such as proteins, nucleic acids, and polysaccharides). This process is crucial for normal cellular function, signal transduction, and metabolic regulation. For example, certain enzymes require localization to specific organelles to perform their catalytic functions, whereas some signaling proteins require localization to the cell membrane to receive and transmit signals. “Metabolic process” is a relatively broad concept at a relatively high level, encompassing numerous specific metabolic processes. For example, “fatty acid metabolic process”, “carboxylic acid metabolic process”, and “amino acid metabolic process” are all subcategories of this term. Our results suggest that disrupted cellular processes and metabolism play significant roles in DIPG, consistent with the subsequent metabolomics findings. In the MF category, the downregulated proteins were enriched primarily in binding functions. “Binding” refers to the ability or function of a biomolecule, such as a protein, to bind to a specific ligand, substrate, or other molecule. These functions include purine ribonucleoside triphosphate binding and purine nucleotide binding. These findings suggest that binding functions are associated with abnormal purine metabolism, potentially providing clues to the pathogenesis of DIPG and potential therapeutic drug targets. The KEGG pathway analysis revealed that upregulated proteins were significantly enriched in the complement and coagulation cascades, suggesting that complement and coagulation cascades may play crucial roles in DIPG. More interestingly, our analysis revealed that downregulated proteins in CSF from patients with DIPG were enriched in numerous infectious pathways, including Yersinia infection, bacterial invasion of epithelial cells, Salmonella infection, pathogenic Escherichia coli infection, shigellosis, and epithelial cell signaling in Helicobacter pylori infection. We initially suspected cerebrospinal fluid contamination, but we collected the cerebrospinal fluid in a completely sterile manner, and we handled the samples according to standard procedures. Currently, no studies have described a direct association between DIPG and pathogenic microbial infection. However, increasing evidence has indicated that the microbiome is strongly correlated with various central nervous system diseases, including brain tumors. Increasing evidence suggests that the tumor-associated microbiome (such as the gut microbiome) may play an integral role in the pathogenesis and pathophysiology of gliomas. Clinical studies have revealed significant changes in the gut microbial community structure in glioblastoma patients, including increases in the abundance of pathogens from the Fusobacteria and Bacteroidetes phyla and decreases in the abundance of probiotics from the Firmicutes, Actinobacteria, and Verrucomicrobia phyla [28]. Studies have also demonstrated that the gut microbiota of patients with recurrent glioblastoma treated with bevacizumab combined with temozolomide differs from that of patients treated with temozolomide alone. The fecal microbiota of patients receiving bevacizumab and temozolomide combined with temozolomide, as well as those receiving temozolomide alone, were primarily composed of Firmicutes, Proteobacteria, Bacteroidetes, and Actinobacteria. However, the relative abundances of Firmicutes, Bacteroidetes, and Actinobacteria were higher in the fecal microbiota of patients in the combination treatment group, whereas the relative abundances of Bacteroidetes and Cyanobacteria were lower [29]. This study contributes to a better understanding of the impact of bevacizumab-related treatment on patients with recurrent glioblastoma. Therefore, these findings may also support potential treatment options for patients with recurrent glioblastoma, such as fecal microbiota transplantation. Traditionally, researchers believed that commensal microorganisms in tumor tissues were present only in tumors that directly interact with the outside world, such as gastrointestinal cancers. However, increasing evidence suggests that microorganisms may also exist in a wider range of cancer types originating from some “sterile” organs. The intratumoral microbiome is a crucial component of the tumor microenvironment, directly interacting with it and further influencing tumor development and progression. With the advancement of molecular biology techniques, information on the intratumoral microbiome has been reported for various tumor types, including GBM. Nejman D et al. documented the presence of an intratumoral microbiome in gliomas using a combination of immunohistochemistry, fluorescence in situ hybridization, electron microscopy, culture-based genomics, and genomic sequencing [30]. Using 16S rRNA gene sequencing, transcriptome sequencing, and untargeted metabolomic analysis, the research team comprehensively analyzed the microbial community composition, gene expression profiles, and metabolic signatures in glioma tissue [31]. The results showed that the microbial community in glioma tissue differed significantly from that in normal brain tissue and that the presence of specific bacteria was closely associated with changes in the expression of certain neuron-related genes in tumor cells. Further mechanistic studies suggest that these bacteria may influence the biological behavior of tumor cells through their metabolites, thereby playing a significant role in the development and progression of gliomas. The origin of the glioma microbiome remains to be elucidated [32]. One possibility is that bacteria may already be present in brain tissue before tumorigenesis, subsequently inducing the initiation and migration of gliomas. Another possibility is that gliomas may alter the local microenvironment, such as by disrupting the blood–brain barrier and cellular barriers, which, combined with relative immunosuppression, allows bacteria to invade tumors from other sites. Furthermore, intestinal bacteria may enter the brain or cross the blood–brain barrier via the vagus nerve that innervates the intestine. Studies have also revealed the presence of intracellular bacteria within tumors, suggesting that these bacteria may enter tumor tissue during the migration of immune cells and cancer cells. However, all of these speculations require further confirmation.

Researchers have also investigated the relationship between diffuse midline gliomas and the microbiome. An international study of the gut microbiome in children with diffuse midline glioma (DMG) is of significant exploratory significance [33]. Based on data from the cohort in the PNOC022 trial, the associations between baseline gut microbial alpha diversity and the progression-free survival (PFS) and overall survival (OS) of DMG patients were identified for the first time. In particular, the group with a high alpha diversity showed a significant advantage in PFS, providing a new perspective for understanding the role of the gut microbiome in this intractable pediatric brain tumor. Our study also provides preliminary evidence for microbiological research on DIPG. However, further in vivo animal and clinical studies are needed to elucidate the regulatory role of the microbiome in the pathogenesis of DIPG.

Moreover, the interactions and mechanisms between metabolites and the central nervous system have received extensive attention. However, the correlations among the microbiome, metabolites, and glioma remain unclear. Glioma metabolites are another area of increasing interest in the field of cerebrospinal fluid biomarkers. Metabolites are various molecules produced during cellular metabolism, including intermediates and end products, many of which have been proposed as potential diagnostic or prognostic biomarkers for glioma growth. Numerous studies have proposed a large number of diagnostic metabolites that can be used to distinguish malignant gliomas from healthy controls. The types of these metabolites indicate certain metabolic pathways that are unique to malignant gliomas. In a study investigating the relationship between 124 metabolites and malignant glioma, the cerebrospinal fluid of 10 patients with malignant glioma and 7 controls was analyzed. Among the 38 metabolites that were significantly elevated in patients with malignant tumors, glucose-1-phosphate, glutamine, and 7-methylguanosine were the most strongly correlated [34]. Another study of 125 CSF metabolites highlighted the importance of carnitine, 2-methylbutyrylcarnitine, and shikimic acid. The first two have been implicated in lipid metabolism in glioblastoma, whereas shikimic acid suggests a potential link between tumors and the gut microbiome [35]. In a study of CSF biomarkers of CNS tumors, interesting associations were observed between IDH-mutant gliomas and IDH-wild-type gliomas. Acetylcarnitine and shikimic acid levels were significantly higher in the CSF of patients with IDH-wild-type gliomas than in those of controls. Malate and succinate (part of the tricarboxylic acid cycle) were present at higher concentrations in IDH-mutant gliomas and may serve as markers to distinguish them from IDH-wild-type gliomas [36]. A new study of low-mass ions (LMIs) in the CSF of 32 patients with primary brain tumors (a total of 10,408 candidate ions) yielded interesting results. This study aimed to distinguish gliomas from nontumor controls, gliomas of different grades, grade 4 glioblastomas (GBMs) and medulloblastomas, and glioma patients with and without leptomeningeal metastases. Patients with grade 3 gliomas had elevated levels of sphingosine and ceramide, whereas in patients with grade 4 gliomas, the highest levels of LMIs were observed for aldose, glycerate, and acetate. A total of 2674 lipid metabolism indices (LMIs) were identified, some of which were able to distinguish glioma patients from controls. Elevated levels of carnitine and phosphate distinguish GBM from medulloblastomas, which are characterized by elevated concentrations of carboxylates and pteridines. Finally, depending on the technique used, many lipid metabolism indices may play a role in revealing the presence of leptomeningeal metastases [37]. Zaeiner et al. investigated the cerebrospinal fluid penetration of the multikinase inhibitor regorafenib in patients with recurrent malignant gliomas to assess its efficacy. Regorafenib is present at low concentrations in cerebrospinal fluid and may induce certain tumor growth patterns, as shown by magnetic resonance imaging (MRI), resulting in a poor overall treatment response. In conclusion, further studies are needed to explore the prognostic relevance of regorafenib, and researchers recommend that it should be used with caution when administered as a last resort [38].

Purine nucleotides are essential for RNA and DNA synthesis, signal transduction, metabolism, and energy homeostasis [39]. The relationships between purine metabolism and the clinicopathological features and immune microenvironment of gliomas have been studied [40]. A study of 1523 tumors from four public databases investigated the relationship between the expression of purine metabolism-related genes (PuMGs) and glioma characteristics, including prognosis and the tumor microenvironment. The results of the analysis indicated that PuMRS was an independent prognostic factor for glioma. Furthermore, an analysis of the tumor immune microenvironment revealed that higher PuMRS levels correlated with increased immune cell infiltration and gene expression signatures in “hot” tumors. This study elucidated the relationship between PuMG expression levels and glioma aggressiveness, demonstrating that the application of PuMRS can lead to the establishment of a new and reliable model for assessing the prognosis of patients with brain tumors. Other studies suggest that purine metabolism may be a new therapeutic target for GBM [41]. Daniel R Wahl et al. reported that purine metabolites mediate radioresistance by promoting DNA repair and that purine synthesis inhibitors can reverse radioresistance in GBM. High expression of *IMPDH*, the rate-limiting enzyme for de novo GTP synthesis, in patients is associated with a poor prognosis. Inhibiting purine synthesis may represent a new approach to enhance the efficacy of glioma radiotherapy. This finding is supported by the literature. GBM stem cells generally exhibit robust de novo nucleotide synthesis and are closely associated with treatment resistance and disease recurrence.

Metabolomic studies have also been conducted on diffuse midline glioma (DMG) [42]. A metabolomic analysis of H3K27M-congenic DMG cell lines, both irradiated and unirradiated, revealed that H3K27M specifically enriched the purine synthesis pathway. DMG-H3K27M cells activated purine metabolism in an H3K27M-specific manner. In the absence of genotoxic treatment, H3K27M-expressing cells presented higher relative de novo synthesis activity and significantly lower purine salvage activity, as demonstrated by stable isotope tracing of key metabolites involved in purine synthesis and reduced expression of hypoxanthine–guanine phosphoribosyltransferase (*HGPRT*), the rate-limiting enzyme for purine salvage conversion to IMP and GMP. The inhibition of de novo guanylate synthesis sensitized DMG-H3K27M cells both in vitro and in vivo. Irradiated H3K27M cells upregulated *HGPRT* expression and hypoxanthine-derived guanylate salvage activity but maintained high levels of guanine-derived salvage activity. Exogenous guanine supplementation reduced the radiosensitization of cells treated with combined radiation therapy and de novo purine synthesis inhibitors. Combining *HGPRT* silencing with radiation therapy significantly inhibited the growth of DMG-H3K27M tumors in vivo. The authors’ results suggest that DMG-H3K27M cells rely on highly active purine synthesis, including both the de novo and salvage pathways. However, efficient salvage of free purine bases to generate mature guanylate can circumvent the inhibition of the de novo synthesis pathway. Our study also revealed that purine metabolism is disrupted in DIPG patients, suggesting that interfering with purine metabolism may be a promising strategy to overcome treatment resistance in DMG-H3K27M tumors.

## 4. Materials and Methods

### 4.1. Biospecimen Collection and Clinical Data

**Figure 8:**
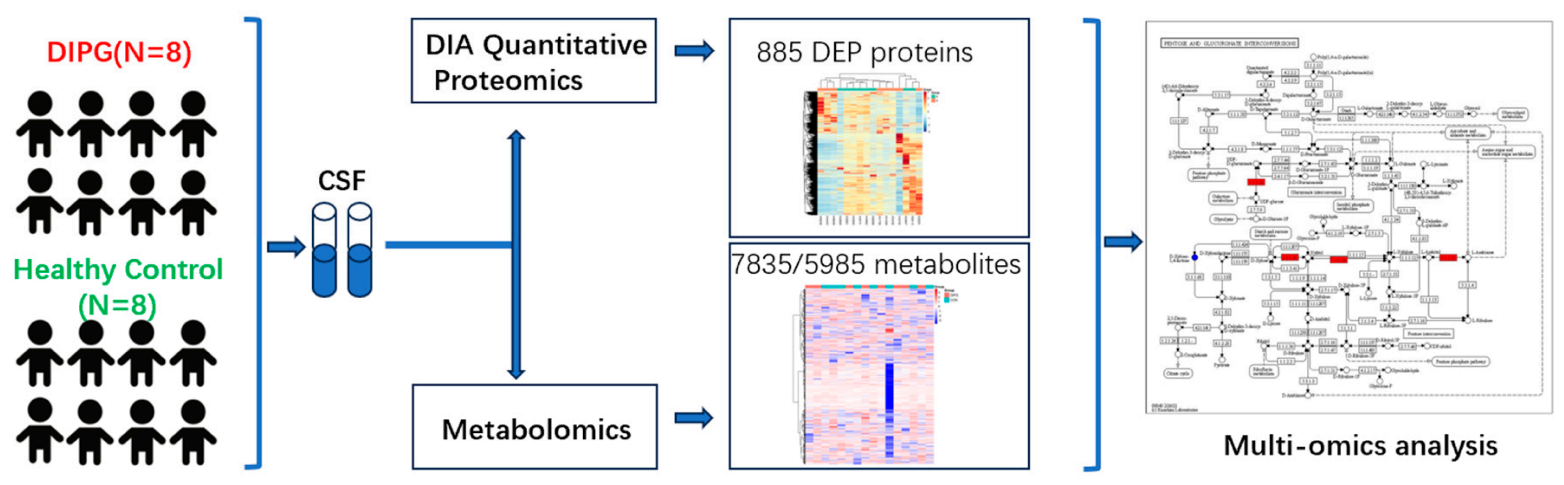
Overview of the study design and patient group allocation. Pediatric patients with DIPG (n = 8) and patients with arachnoid cysts were included in this study.

In this study, pediatric patients with DIPGs admitted to the Department of Pediatric Neurosurgery at Xinhua Hospital Affiliated to Shanghai Jiao Tong University School of Medicine were enrolled. Eight age- and sex-matched patients with arachnoid cysts served as the control group. The basic clinical characteristics of all the patients are shown in Table 6. The imaging features of all DIPG patients are shown in Supplementary Figure S8. Cerebrospinal fluid samples were collected from all patients for proteomic and metabolomic analyses, and the study workflow is shown in Figure 8. DIPG was diagnosed based on the clinical features, imaging data, and pathological findings. All patients underwent lumbar puncture before surgery, and 5 mL of cerebrospinal fluid was obtained. All cerebrospinal fluid was free of blood. Within 30 min, the samples were centrifuged at 1000× *g* for 10 min and stored at −80 °C. Metabolomic and proteomic data were collected simultaneously from the same biospecimen and integrated for analysis to provide a comprehensive proteomic and metabolomic profile of DIPG. This study was approved by the Institutional Review Board of Xinhua Hospital, affiliated with Shanghai Jiao Tong University School of Medicine, and informed consent was obtained from the patients’ parents.

### 4.2. Proteomics

#### 4.2.1. Basic Methods

In this study, DIA (data-independent acquisition) quantitative proteomics was employed, with the following key steps: protein extraction, protein quantification, protein digestion, LC-MS/MS analysis, database search, and data analysis. Sample preparation: After freeze-drying, an appropriate amount of SDT lysis buffer was added to each sample, which was subsequently transferred to an EP tube. The sample was then boiled in a water bath for 3 min, sonicated for 2 min, and centrifuged at 16,000× *g* for 20 min at 4 °C. The supernatant was collected, and the protein concentration was quantified using a BCA assay. An appropriate amount of protein from each sample was digested using FASP. The resulting peptides were dried and reconstituted in 0.1% FA. Peptide concentrations were determined for the LC-MS analysis. Approximately 300 ng of peptides from each sample was chromatographically separated using a Vanquish Neo UHPLC system (Thermo Scientific, Waltham, MA, USA). Buffer solutions: Solution A was a 0.1% formic acid aqueous solution, and Solution B was a 0.1% formic acid–acetonitrile aqueous solution (acetonitrile content 80%). The chromatographic column was equilibrated with 96% of Solution A. After the sample was injected into the trap column (PepMap Neo 5 µm C18 300 µm × 5 mm, Thermo Scientific), it underwent gradient separation using the analytical column (μPAC Neo High-Throughput column, Thermo Scientific). The HPLC gradient settings were as follows: 0 min–0.1 min, linear gradient of Solution B from 4% to 6%; 0.1 min–1.1 min, linear gradient of Solution B from 6% to 12%; 1.1 min–4.3 min, linear gradient of Solution B from 12% to 22.5%; 4.3 min–6.1 min, linear gradient of Solution B from 22.5% to 45%; and 6.1 min–8 min, Solution B was maintained at 99%. After peptide separation, DIA (data-independent acquisition) mass spectrometry analysis was performed using an Orbitrap Astral mass spectrometer (Thermo Scientific). The analysis time was 8 min, the electrospray voltage was 2.2 kV, the detection mode was positive ion, the precursor ion scan range was 380–980 *m*/*z*, the first-stage mass spectrometry resolution was 240,000, the AGC target was 500%, and the first-stage maximum IT was 3 ms. The second-stage mass spectrometry resolution was 80,000, the AGC target was 500%, the second-stage maximum IT was 3 ms, the RF lens was 40%, the MS2 activation type was HCD, the isolation window was 2 Th, the normalized collision energy was 25%, and the cycle time was 0.6. All mass spectrometry data were merged using DIA-NN software(version 1.8.1) to perform database searches and quantify the proteins identified in DIA mode. The resulting DIA raw mass spectrum files were imported into DIA-NN for DIA analysis, using a Q value ≤ 0.01 as the filtering parameter.

#### 4.2.2. Data Quality Control and Analysis

In this experiment, a high-quality, high-resolution mass spectrometer was used to maintain low mass deviation during data acquisition. The peptide mass error threshold was set at ±10 ppm. Combined with the library search qualitative thresholds of a peptide FDR ≤ 0.01 and a protein FDR ≤ 0.01 as filtering criteria, along with the peptide score distribution, this analysis produced high-quality data for subsequent analysis and accurate and reliable identification results. Based on protein expression across samples, expression distribution, analysis, correlation analysis, and PCA were performed. The results were used to assess intergroup variability and intragroup reproducibility from various perspectives. For the analysis of significant differences in protein levels, Student’s *t*-test was used, and the *p*-value + FC method was used to screen for significantly differentially expressed proteins. If screening thresholds were used, the *p*-value screening threshold was <0.05, the FC threshold was ≥1.5 or ≤1/1.5, and the VIP (OPLS-DA) threshold was >1. Proteins meeting the screening criteria of a fold difference greater than 1.5 (up- or downregulated) and a *p*-value less than 0.05 were considered significantly differentially expressed. The subcellular localization of the differentially expressed proteins was annotated and statistically analyzed by determining the cellular component (CC) terms in the Gene Ontology (GO) database. Differentially expressed proteins (DEPs) were submitted to the DAVID website (https://davidbioinformatics.nih.gov/, accessed on 1 May 2024) for functional annotation. Biological process, cellular component, and molecular function terms were analyzed using the Gene Ontology (GO) database (http://amigo.geneontology.org, accessed on 1 June 2024). The functional pathway analysis was performed using the Kyoto Encyclopedia of Genes and Genomes (KEGG) database (http://www.kegg.jp/, accessed on 1 June 2024). The hypergeometric distribution test was used to determine the significance of enriched DEPs within each GO term or KEGG pathway. The results were screened using a pathway enrichment test with a *p*-value less than 0.05 and a protein count greater than 5.

### 4.3. Metabolomics

#### 4.3.1. Metabolite Extraction and LC-MS Analysis

Samples were thawed at 4 °C, vortexed for 1 min, and then extracted with 300 μL of methanol–acetonitrile (2:1, *v*/*v*) by vortexing for 1 min and sonication in ice for 10 min. After centrifugation at 12,000 rpm for 10 min at 4 °C, 300 μL of supernatant was collected, dried, and reconstituted in 150 μL of methanol–water (1:4 *v*/*v*) containing 2-chloro-L-phenylalanine as an internal standard. The resulting solution was filtered through a 0.22 μm membrane prior to LC-MS analysis. Chromatographic separation was performed on an ACQUITY UPLC^®^ HSS T3 column (2.1 × 100 mm, 1.8 µm) using a Thermo Ultimate 3000 system (Thermo Fisher Scientific, Waltham, MD, USA). The flow rate was 0.3 mL/min, the column temperature was 40 °C, and the injection volume was 2 μL. For positive ion mode, the mobile phases were 0.1% formic acid in water (A) and 0.1% formic acid in acetonitrile (B). For negative ion mode, 5 mM ammonium formate in water (C) and acetonitrile (D) were used. The gradient program for both modes was 0–1 min, 10% B/D; 1–7.5 min, 10–98% B/D; 7.5–10 min, 98% B/D; 10–10.1 min, 98–10% B/D; and 10.1–12 min, 10% B/D. Mass spectrometry was carried out on a Thermo Q Exactive instrument (Thermo Fisher Scientific, USA) with an electrospray ionization (ESI) source in both positive and negative modes. The spray voltages were set at 3.50 kV (positive) and −2.50 kV (negative), with sheath gas at 40 arb and auxiliary gas at 10 arb; the capillary temperature was 325 °C. Full-scan MS data were acquired at a resolution of 70,000 over an *m*/*z* range of 81~1000, followed by HCD fragmentation (collision energy 30 eV) of the top 10 precursor ions at a resolution of 17,500, with dynamic exclusion applied to minimize redundant MS/MS acquisition.

#### 4.3.2. Data Preprocessing

The metabolomic analysis was performed using liquid chromatography–tandem mass spectrometry (LC-MS/MS). First, the raw mass spectrometry files were converted to mzXML format using the MSConvert tool in the ProteoWizard package (v3.0.8789). Peak detection, peak filtering, and peak alignment were performed using the RXCMS software package (v3.12.0) to generate a metabolite quantification list. The parameters were bw = 2, ppm = 15, peak width = c (5, 30), mzwid = 0.015, mzdiff = 0.01, and method = “centWave”. Support vector regression correction based on the QC samples was then performed to eliminate systematic errors. During the quality control and quality assurance process, compounds with an RSD > 30% in the QC samples were filtered out for subsequent data analysis. Substances were identified using spectral databases, including public databases such as HMDB, MassBank, LipidMaps, mzcloud, and KEGG, as well as a self-built standard library. The parameters were set to <30 ppm to obtain qualitative data for the metabolites. The molecular weight of the metabolite was determined based on the mass-to-charge ratio (*m*/*z*) of the precursor ion in the primary mass spectrum. Molecular formulas were then predicted using information such as mass deviation (ppm) and adduct ions. These predicted formulas were then matched to those in the database. Simultaneously, metabolites detected in the secondary spectra were matched to fragment ions and other information in the quantification list in the database to achieve the identification of secondary metabolites.

#### 4.3.3. Data Analysis

The sample data were analyzed using the R software package Ropls (version 1.38.0) for dimensionality reduction with principal component analysis (PCA), partial least squares discriminant analysis (PLS-DA), and orthogonal partial least squares discriminant analysis (OPLS-DA). The model was tested for overfitting using a permutation test. R2X and R2Y represent the explanatory power of the model for the X and Y matrices, respectively, whereas Q2 indicates the predictive power of the model. The closer their values are to 1, the better the model fit and the more accurately the training set samples are classified into their original categories. *p*-values were calculated using statistical tests (*t*-test algorithm: applicable to experimental designs in which each sample group includes at least three biological replicates or mass spectrometry replicates; significance A algorithm: applicable to experimental designs in which each sample group has fewer than three biological replicates or mass spectrometry replicates). Variable projection importance (VIP) was calculated using the OPLS-DA dimensionality reduction method, and the fold change was calculated using the intergroup difference factor to measure the impact and explanatory power of the content of each metabolite on sample classification, assisting in the screening of marker metabolites. Metabolites were considered significantly differentially expressed when the *p*-value was <0.05 and the VIP value was >1.

#### 4.3.4. Pathway Analysis

A pathway enrichment analysis was performed using a hypergeometric distribution-based enrichment analysis. The enriched pathways were visualized using the KEGG Mapper tool (version 5.2) to visualize differentially abundant metabolites and pathway maps.

#### 4.3.5. Integrated Analysis

Multiomics association analysis mainly includes cross-omics association analysis, combined biomarker discovery based on machine learning algorithms, and in-depth mining of multiomics data. Among them, the cross-omics association analysis currently consists of four parts: (1) an association analysis based on references and databases; (2) association analysis based on metabolic pathway analysis; (3) association analysis based on interactions; and (4) association analysis based on statistical methods. The association analysis based on statistical methods includes not only a correlation-based integrated analysis, such as Pearson’s correlation analysis and Spearman’s rank correlation analysis, but also an integrated analysis based on data splicing, an integrated analysis based on a multivariate model, and an integrated analysis based on metabolic pathways.

#### 4.3.6. Molecular Docking

To functionally validate the multiomics findings and explore potential direct interactions between significantly altered proteins and key metabolites, we selected three differentially expressed proteins (URB1, RALBP1, and JPT2) and three crucial metabolites (sulfate, hypoxanthine, and xanthosine) from the enriched purine and tyrosine metabolism pathways for molecular docking. The predicted structures of the receptor proteins were generated via AlphaFold (version 2.3.2). The protonation state of all the compounds was set at pH = 7.4, and the compounds were expanded to 3D structures using Open Babel. AutoDock Tools (ADT3) were applied to prepare and parametrize the receptor protein and ligands. The docking grid documents were generated by AutoGrid of sitemap, and AutoDock Vina (1.2.0) was used for docking simulation. The optimal pose was selected to analyze the interaction. Finally, the protein–ligand interaction figure was generated by PyMOL (version 2.0).

## 5. Conclusions

Overall, this study presents the first comprehensive integrated proteomic and metabolomic analyses of pediatric patients with DIPG using the same cerebrospinal fluid samples. The identified signature proteins and metabolites form a complex network and delineate key immunometabolic processes associated with DIPG. Despite the relatively small sample size, our analyses yielded promising data that advance our understanding of DIPG pathogenesis and guide the identification of molecular mechanisms and the development of targeted therapies for this devastating disease. The combined use of proteomics and metabolomics provides new insights that are not achievable by single-omic analyses.

## Figures and Tables

**Table 1:** The top 20 upregulated proteins ranked by fold change.

Accession	Gene Symbol	Protein Name	Mean.A	Mean.B	A/B
A0A5C2G514		IGL c1393_light_IGLV1-47_IGLJ3 (Fragment)	26,708.2756	207.207988	128.895975
A0A5C2GTY0		IG c49_light_IGKV1D-13_IGKJ5 (Fragment)	7663.82076	360.250011	21.273617
A0A5C2GKA1		IGH + IGL c447_light_IGLV3-25_IGLJ3 (Fragment)	2813.82134	157.651607	17.8483518
A0A5C2FTW2		IGL c152_light_IGKV1D-13_IGKJ5 (Fragment)	3915.9658	224.992617	17.4048636
A0A5C2G138		IGL c3008_light_IGLV9-49_IGLJ3 (Fragment)	11,449.5435	950.781728	12.0422418
A0A5C2GPH9		IG c594_heavy_IGHV1-69_IGHD2-2_IGHJ4 (Fragment)	3838.72153	322.62324	11.8984656
A0A7S5C0A4		IGH c1813_heavy_IGHV4-34_IGHD3-22_IGHJ4 (Fragment)	21,945.4878	2001.477	10.9646465
A0A5C2FZB2		IGL c1580_light_IGKV3-20_IGKJ4 (Fragment)	21,350.7745	2870.84386	7.43710754
O60287	URB1	Nucleolar pre-ribosomal-associated protein 1	13,200.1888	1890.10442	6.98384106
O95633	FSTL3	Follistatin-related protein 3	3556.61858	587.140527	6.05752528
A0A5C2GL52		IG c1102_light_IGKV1-9_IGKJ3 (Fragment)	6191.05258	1065.25607	5.8117975
A0A5C2GTA2		IG c1297_heavy_IGHV4-39_IGHD3-16_IGHJ6 (Fragment)	5569.19625	972.019819	5.72950895
P25713	MT3	Metallothionein-3	2766.53156	501.460502	5.51694809
A0A5C2FU04		IGL c441_light_IGKV3D-15_IGKJ1 (Fragment)	24,929.6219	4521.83015	5.51317079
A0A5C2G7M0		IGL c2403_light_IGKV3-20_IGKJ1 (Fragment)	10,940.9507	2432.87575	4.49712677
A0A5C2GFX4		IGH + IGL c71_light_IGKV1-9_IGKJ2 (Fragment)	18,629.722	4176.59713	4.46050251
P29762	CRABP1	Cellular retinoic acid-binding protein 1	22,571.8324	5173.81516	4.36270561
A0A5C2GJP7		IG c399_light_IGKV2D-29_IGKJ3 (Fragment)	41,108.9504	10,006.2763	4.10831655
A0A5C2FW64		IGL c1328_light_IGLV2-23_IGLJ1 (Fragment)	10,723.403	2683.1512	3.99657055
A0A5C2GMA8		IG c30_light_IGLV2-11_IGLJ2 (Fragment)	9895.78357	2476.52595	3.99583279

A: DIPG Group; B: Con Group.

**Table 2:** The top 20 downregulated proteins ranked by fold change.

Accession	Gene Symbol	Protein Name	Mean.A	Mean.B	A/B
O00160	MYO1F	Unconventional myosin-If	57.99992	9693.37814	167.12744
O75441		Latent transforming growth factor-beta binding protein 4 (Fragment)	57.99992	9198.39819	158.593291
A0A5C2GHU5		IGH + IGL c315_heavy_IGHV3-11_IGHD3-3_IGHJ2 (Fragment)	57.99992	8302.28681	143.143073
Q9NR48	ASH1L	Histone-lysine N-methyltransferase ASH1L	57.99992	5015.05175	86.4665287
P56945	BCAR1	Breast cancer anti-estrogen resistance protein 1	57.99992	4628.28728	79.7981665
A0A858B990	HLA-A	MHC class I antigen	57.99992	4469.64407	77.062935
O75150	RNF40	E3 ubiquitin-protein ligase BRE1B	57.99992	4378.34542	75.4888182
Q8IXL7	MSRB3	Methionine-R-sulfoxide reductase B3	57.99992	4036.32201	69.5918547
Q6XE24	RBMS3	RNA-binding motif, single-stranded-interacting protein 3	57.99992	3860.92488	66.5677621
C4AMC7	WASH3P	Putative WAS protein family homolog 3	57.99992	3620.09857	62.4155787
Q6IA17	SIGIRR	Single Ig IL-1-related receptor	57.99992	3327.13924	57.3645488
Q9BUI4	POLR3C	DNA-directed RNA polymerase III subunit RPC3	57.99992	3249.47751	56.0255515
Q08830	FGL1	Fibrinogen-like protein 1	57.99992	3218.74456	55.4956723
Q9H4A5	GOLPH3L	Golgi phosphoprotein 3-like	57.99992	3097.39712	53.4034723
Q9H4B0	OSGEPL1	tRNA N6-adenosine threonylcarbamoyltransferase, mitochondrial	57.99992	3030.72919	52.2540236
Q9BQF6	SENP7	Sentrin-specific protease 7	57.99992	3010.11038	51.8985263
Q15418	RPS6KA1	Ribosomal protein S6 kinase alpha-1	57.99992	2753.4558	47.4734412
Q9UHY1	NRBP1	Nuclear receptor-binding protein	57.99992	2684.07216	46.27717
Q14289	PTK2B	Protein-tyrosine kinase 2-beta	57.99992	2650.86313	45.7045998
Q9BTE3	MCMBP	Mini-chromosome maintenance complex-binding protein	57.99992	2541.56424	43.8201336

A: DIPG Group; B: Con Group.

**Table 3:** List of upregulated differentially expressed metabolites.

Name	mz	rt	FC	*p*-Value	Posneg
Albaflavenone	241.1544	109.5	7.14	0.011611	pos
Cyclooctat-9-en-7-ol	351.2505	593.4	4.47	0.030332	neg
Coniferyl alcohol	241.0745	100.2	4.45	0.024277	neg
Sophoraflavanone G	381.2052	522.9	3.11	0.045251	pos
(4-Chloro-2-methylphenoxy)acetic acid	199.1696	435.3	3.04	8.53 × 10^−5^	neg
Methoxamine	194.1169	76	2.77	0.006823	pos
9Z-Octadecenedioic acid	311.2223	413.4	2.4	0.008306	neg
3,4-Dihydroxymandelic acid	141.0529	205.8	2.27	0.014892	pos
Protomycinolide IV	366.2594	376.9	2.26	0.026216	pos
2-Propenoic acid, 3-(2,5-dihydroxyphenyl)-	163.9555	679.5	2.17	0.026297	pos
Sequoyitol	151.096	223	1.97	0.007823	pos
Pirimicarb	261.137	405.6	1.92	0.015357	pos
cis-1,2-Dihydroxy-1,2-dihydrodibenzothiophene	217.0289	53.4	1.86	0.019335	neg
Hypoxanthine	137.0455	35.8	1.81	0.026377	pos
Dihydrocaffeic acid 3-sulfate	245.193	363.9	1.75	0.023938	pos
7-hydroxy-4-isopropenyl-7-methyloxepan-2-one	141.1271	286.7	1.72	0.016543	pos
D-Xylono-1,4-lactone	166.0718	85.5	1.67	0.012703	pos
Xanthosine	283.0677	194.6	1.65	0.021835	neg
1,6-Pyrenediol	273.1659	327	1.64	0.027099	pos
3,3′-Dimethoxybenzidine	245.0777	360.7	1.38	0.025566	pos
4-Methylumbelliferone sulfate	255.2306	43.1	1.34	0.036556	neg
Dialuric acid	183.1165	363.8	1.33	0.023845	pos
2,6-Dihydroxyphenylacetate	169.0492	308.3	1.26	0.016318	pos
Aminoadipic acid	144.0654	189.9	1.22	0.029811	pos

mz, mass-to-charge ratio; rt, retention time, unit: s; FC = mean (experimental group)/mean (control group).

**Table 4:** List of downregulated differentially expressed metabolites.

Name	mz	rt	FC	*p*-Value	Posneg
Sinapic acid	181.0852	309.1	0.9	0.040122	pos
Sulfate	137.0589	34.3	0.84	0.005147	pos
Tabtoxinine-delta-lactam	211.0682	73.9	0.79	0.04931	pos
Sinapine	267.1791	265.3	0.76	0.031163	pos
3,5,5-Trimethyl-2-cyclohexen-1-one	139.1121	335.1	0.66	0.029978	pos
DG(16_1(9Z)_18_0_0_0)	617.5232	428.3	0.66	0.024419	pos
L-Glutamic gamma-semialdehyde	130.0499	95.6	0.64	0.020468	neg
Behenic acid	358.3648	409.2	0.6	0.047312	pos
all-trans-Hexaprenyl diphosphate	604.3643	322	0.51	0.025959	pos
Hydroquinone	128.0705	224.6	0.49	0.040912	pos
Octadecanamide	284.2936	552.5	0.46	0.021683	pos
DG(16_0_0_0_18_2n6)	624.5447	62.2	0.44	0.033202	pos
8,8a-Deoxyoleandolide	355.2497	628.9	0.42	0.02545	pos
pregn-5-ene-3,20-dione	375.2142	435.1	0.41	0.045042	neg
Dibutyl phthalate	279.1582	428.3	0.39	0.027244	pos
3-(Methylthio)-1-propanol	167.0373	363.7	0.31	0.042451	neg

mz, mass-to-charge ratio; rt, retention time, unit: s; FC = mean (experimental group)/mean (control group).

**Table 5:** List of enriched metabolic pathways of differential metabolites.

Pathway	Hits	Up_Hits	Down_Hits	*p*-Value	−Log10(*p* Value)	Compound_Name
Purine metabolism	3	2	1	0.06663797	1.17627823823912	Sulfate; Hypoxanthine; Xanthosine
Tyrosine metabolism	2	1	1	0.16420766	0.784606593314781	Hydroquinone; 3,4-Dihydroxymandelic acid
Caffeine metabolism	1	1	0	0.18704197	0.72806093076498	Xanthosine
Sulfur metabolism	1	0	1	0.2673616	0.572900960570123	Sulfate
Terpenoid backbone biosynthesis	1	0	1	0.35239127	0.452974860408508	all-trans-Hexaprenyl diphosphate
Phenylalanine metabolism	1	1	0	0.37060936	0.431083618642835	2,6-Dihydroxyphenylacetate
ABC transporters	2	1	1	0.37512879	0.425819601787037	Sulfate; Xanthosine
Lysine degradation	1	1	0	0.41120663	0.385939888338763	Aminoadipic acid
Chemical carcinogenesis reactive oxygen species	1	0	1	0.41679484	0.380077670515169	Hydroquinone
Pentose and glucuronate interconversions	1	1	0	0.42781737	0.368741590711835	D-Xylono-1,4-lactone
Cysteine and methionine metabolism	1	0	1	0.46992183	0.327974383823518	Sulfate
Arginine and proline metabolism	1	0	1	0.47996903	0.318786784387088	L-Glutamic gamma-semialdehyde
Biosynthesis of unsaturated fatty acids	1	0	1	0.50428585	0.297323214572187	Behenic acid

Hits: the total number of differential metabolites in the target metabolic pathway; Up_hits: the number of upregulated differential metabolites in the target metabolic pathway; Down_hits: the number of downregulated differential metabolites in the target metabolic pathway.

**Table 6:** Demographics and baseline characteristics of patients.

Group	No.	Gender	Age (Years)	Diagnosis	H3K27M Mutation	Hydrocephalus
Con group	Case 1	Male	5	AC	N/A	(−)
	Case 2	Female	7	AC	N/A	(−)
	Case 3	Male	6	AC	N/A	(−)
	Case 4	Male	6	AC	N/A	(−)
	Case 5	Female	8	AC	N/A	(−)
	Case 6	Female	7	AC	N/A	(−)
	Case 7	Female	4	AC	N/A	(−)
	Case 8	Male	5	AC	N/A	(−)
			Mean: 6.0			
DIPG group	Case 1	Female	2	DIPG	H3K27M(+)	(+)
	Case 2	Male	8	DIPG	H3K27M(+)	(−)
	Case 3	Male	5	DIPG	H3K27M(+)	(−)
	Case 4	Male	4	DIPG	H3K27M(+)	(−)
	Case 5	Male	5	DIPG	H3K27M(+)	(−)
	Case 6	Female	5	DIPG	H3K27M(+)	(−)
	Case 7	Female	5	DIPG	H3K27M(+)	(−)
	Case 8	Female	8	DIPG	H3K27M(+)	(−)
			Mean: 5.3			
			*p*-value: 0.387			

AC: arachnoid cyst; DIPG: diffuse intrinsic pontine glioma; N/A: not available.

## Data Availability

The original contributions presented in this study are included in the article/Supplementary Materials. Further inquiries can be directed to the corresponding author.

## Supplementary Materials

The following supporting information can be downloaded at https://www.mdpi.com/article/10.3390/ijms27156688/s1.
