# Supplementary material for: Integrated Proteomic and Metabolomic Analyses of Cerebrospinal Fluid from Pediatric Patients with Diffuse Intrinsic Pontine Glioma
Source: Int J Mol Sci. 2026 Jul 27;27(15):6688. doi: 10.3390/ijms27156688 (PMC13467317; doi:10.3390/ijms27156688)
Supplement: Supplementary file 1 [file ijms-27-06688-s001.zip › ijms-4352553-supplementary.pdf]

Supplementary Figure S1. A bar chart showing the number of differentially expressed proteins enriched in GO terms.The horizontal axis represents the GO term, the left vertical axis represents the count (number of differentially expressed proteins annotated to a particular term), and the right vertical axis represents the percentage (number of differentially expressed proteins annotated to a particular term/total number of differentially expressed proteins enriched in GO functions). Three major branches, biological processes (BPs), molecular functions (MFs), and cellular components (CCs), are represented by different colors. The specific count is indicated above each column. (A) Downregulated DEPs. (B) Upregulated DEPs.

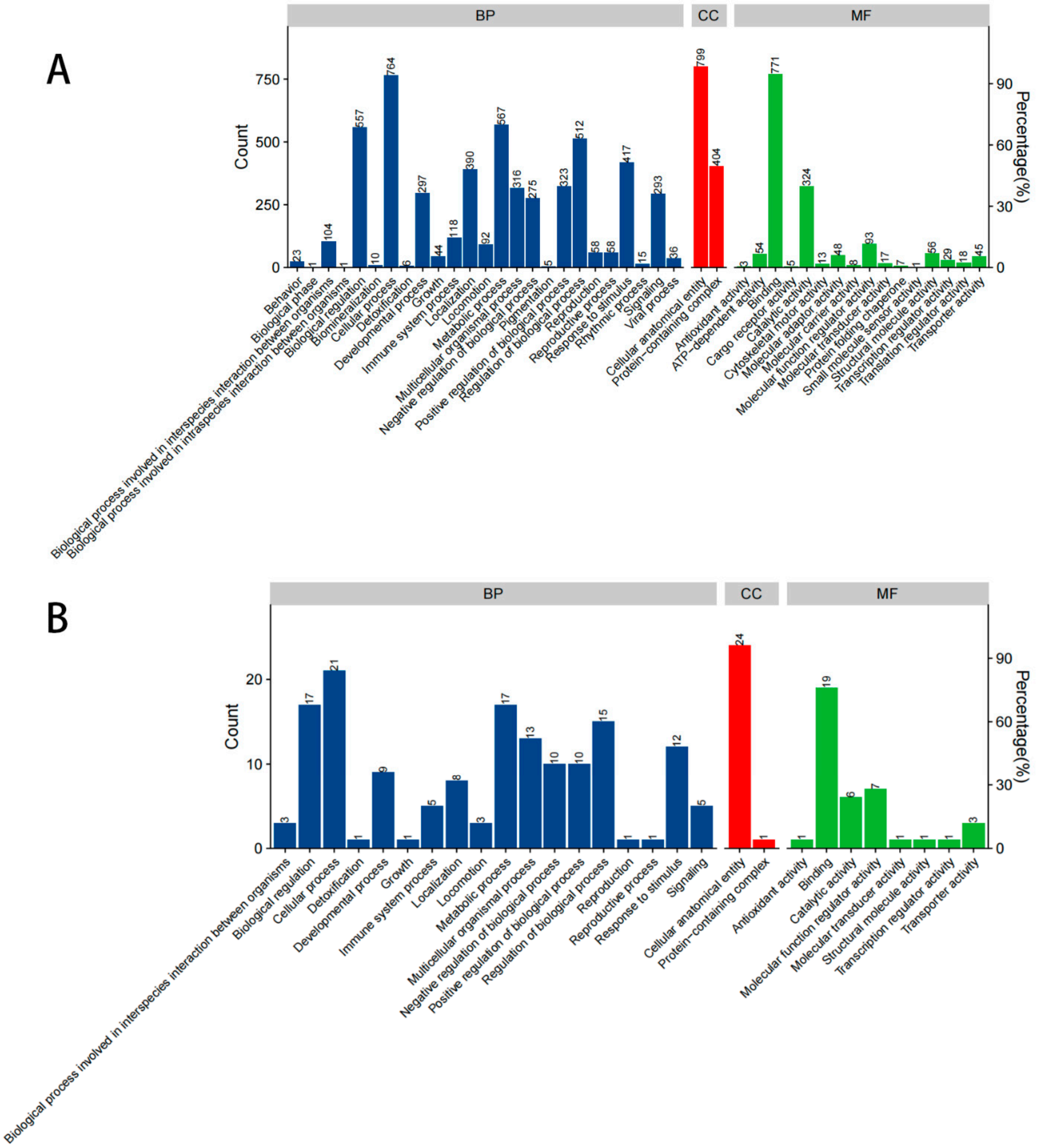

Supplementary Figure S2. GO analysis of the downregulated proteins using all genes in the species.(A) Biological process, BP. (B) Cellular component, CC. (C) Molecular function, MF.

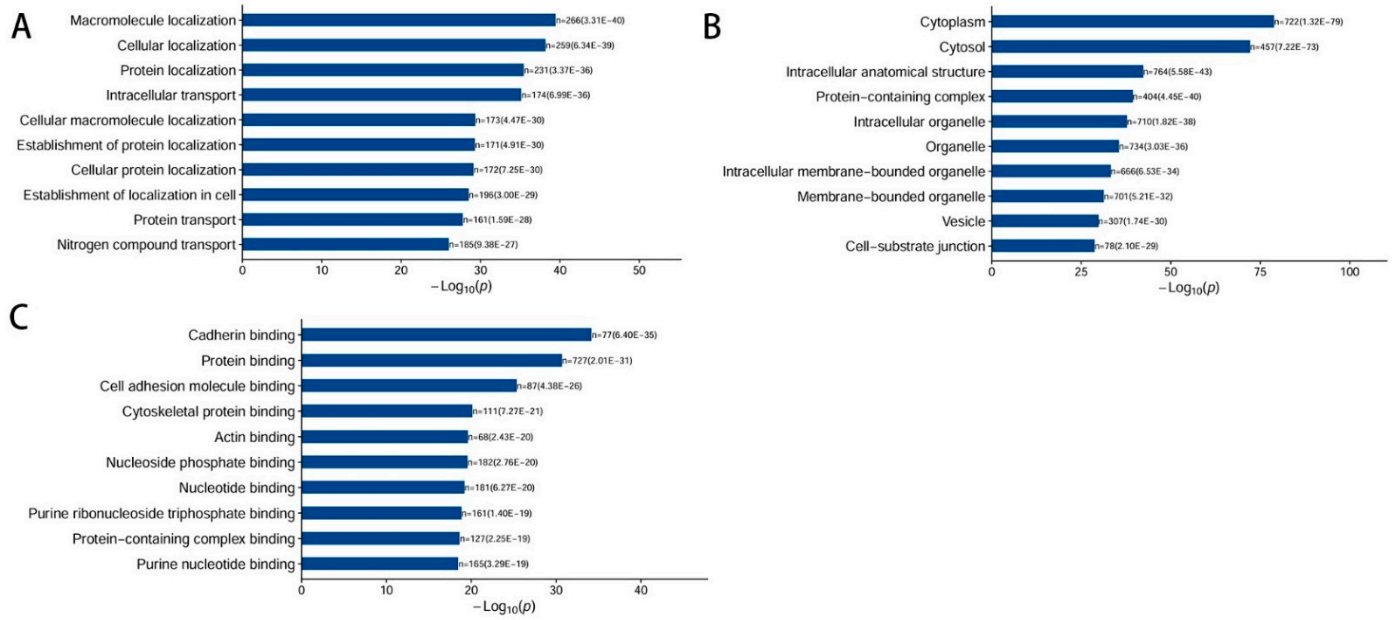

Supplementary Figure S3. Differentially expressed metabolites (DEMs) between the DIPG and Con groups detected in positive ion mode.(A)Scatter plot of differentially abundant metabolite mass-to-charge ratios and P values. The horizontal axis represents the mass-to-charge ratio of a metabolite; the vertical axis represents the -log10 logarithm of the P value. Each dot in the plot represents a metabolite. Red dots represent differential upregulation, blue dots represent differential downregulation, and gray dots represent metabolites that do not meet the differential screening criteria. The dot size represents the VIP value. When three or more groups are compared, no FC information is available; in this case, the differentially abundant metabolites are represented by red dots. By default, the mz values of the top five metabolites with the lowest P values are displayed. (B) Histogram of differentially abundant metabolite statistics. The X-axis represents the number of differentially abundant metabolites, and the Y-axis represents the comparison group. Red indicates the number of upregulated metabolites, and blue indicates the number of downregulated metabolites. (C) Heatmap of differentially abundant metabolite clusters. Columns represent samples, rows represent metabolites, the cluster tree on the left is the differentially abundant metabolite cluster tree, and the top is the sample cluster tree. The gradient color indicates the magnitude of the quantitative value: redder colors indicate higher expression levels, and bluer colors indicate lower expression levels. Metabolite names are not displayed for metabolites with more than 150 metabolites. (D) Volcano plot of differentially abundant metabolites. The horizontal axis represents the log2 of the fold difference in the quantitative expression of a metabolite between the two samples; the vertical axis represents the -log10 of the P value. Each dot in the graph represents a metabolite. A larger absolute value on the horizontal axis indicates a greater fold difference in expression between the two samples; a larger value on the vertical axis indicates a more significant differential expression and a more reliable differentially expressed metabolite. The size of the dot indicates the VIP value. Red dots represent differential upregulation, blue dots represent differential downregulation, and gray dots indicate metabolites that do not meet the differential screening criteria. By default, the mz values of the top five metabolites with the lowest P values are displayed.

https://doi.org/10.3390/xxxxx

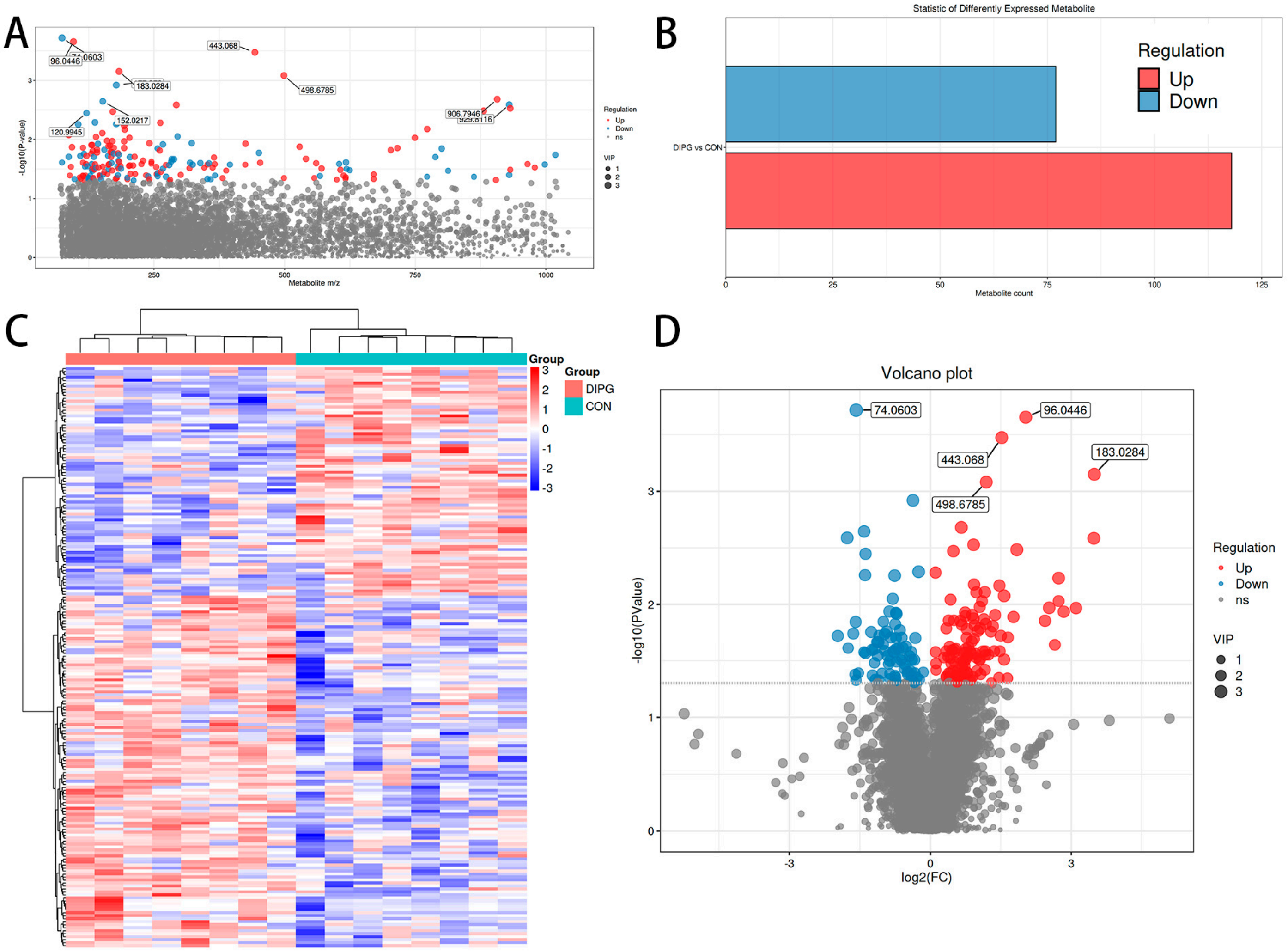

Supplementary Figure S4. Differentially expressed metabolites (DEMs) between the DIPG and Con groups detected in negative ion mode.(A) Scatter plot of differentially abundant metabolite charge ratios and P values. (B) Statistical histogram of differentially abundant metabolites. (C) Cluster heatmap of differentially abundant metabolites. (D) Volcano plot of differentially abundant metabolites.

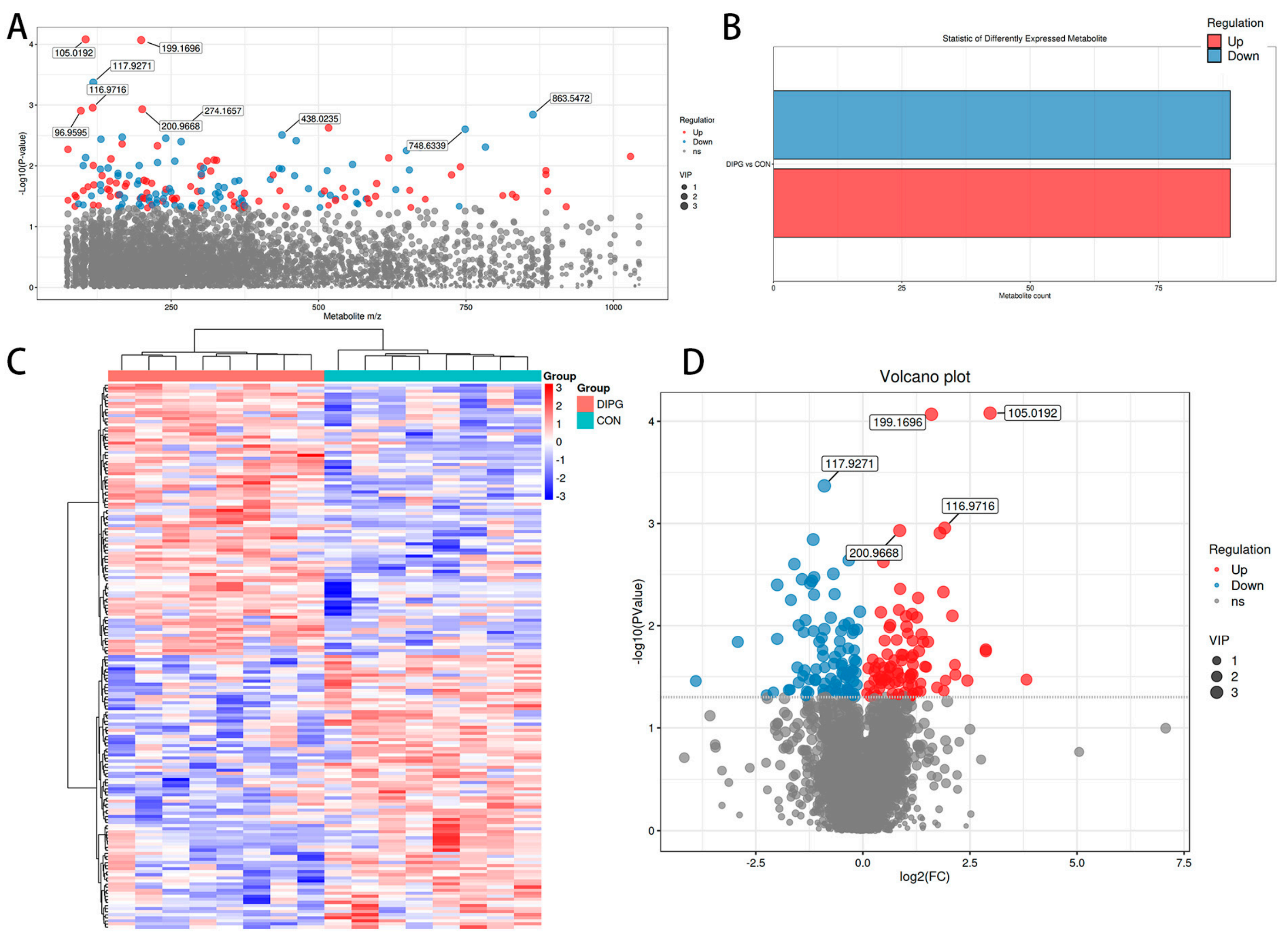

Supplementary Figure S5. Construction of regulatory networks for differentially expressed proteins and metabolites. We simultaneously constructed regulatory networks for the differentially expressed metabolites and proteins using the Metscape plug-in in Cytoscape. This network, which is based on existing knowledge from databases, experiments, and the literature, tracks connections between metabolites and genes, visualizes compound networks, and displays compound structures, reaction types, enzymes, genes, and pathway information. This analysis also enables the verification of differentially expressed proteins at a metabolic level closer to the phenotype.

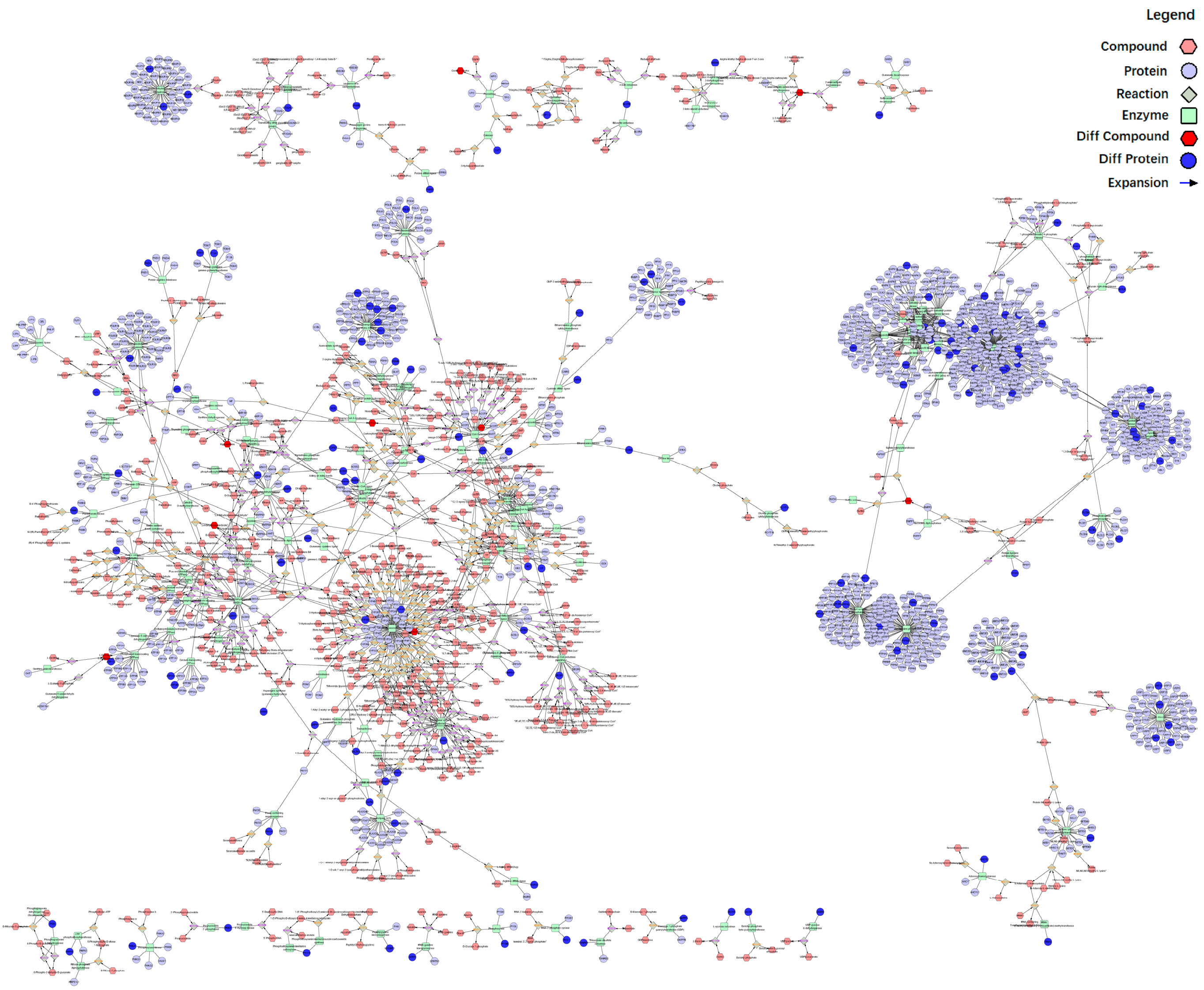

Supplementary Figure S6. Molecular docking analysis of RALBP1 interactions with Sulfate, Hypoxanthine, and Xanthosine. For RALBP1-Sulfate (A), a weak interaction is formed via LEU255 with a binding energy of −2.6 kcal/mol. For RALBP1-Hypoxanthine (B), the complex is stabilized by hydrogen bonds involving ASP218, LEU255, and LYS222, with a binding energy of −4.4 kcal/mol. For RALBP1-Xanthosine (C), the strongest affinity among the three metabolites is observed (binding energy: −5.4 kcal/mol), supported by an extensive hydrogen-bonding network with LYS222, LYS378, VAL376, PRO379, and ARG381.

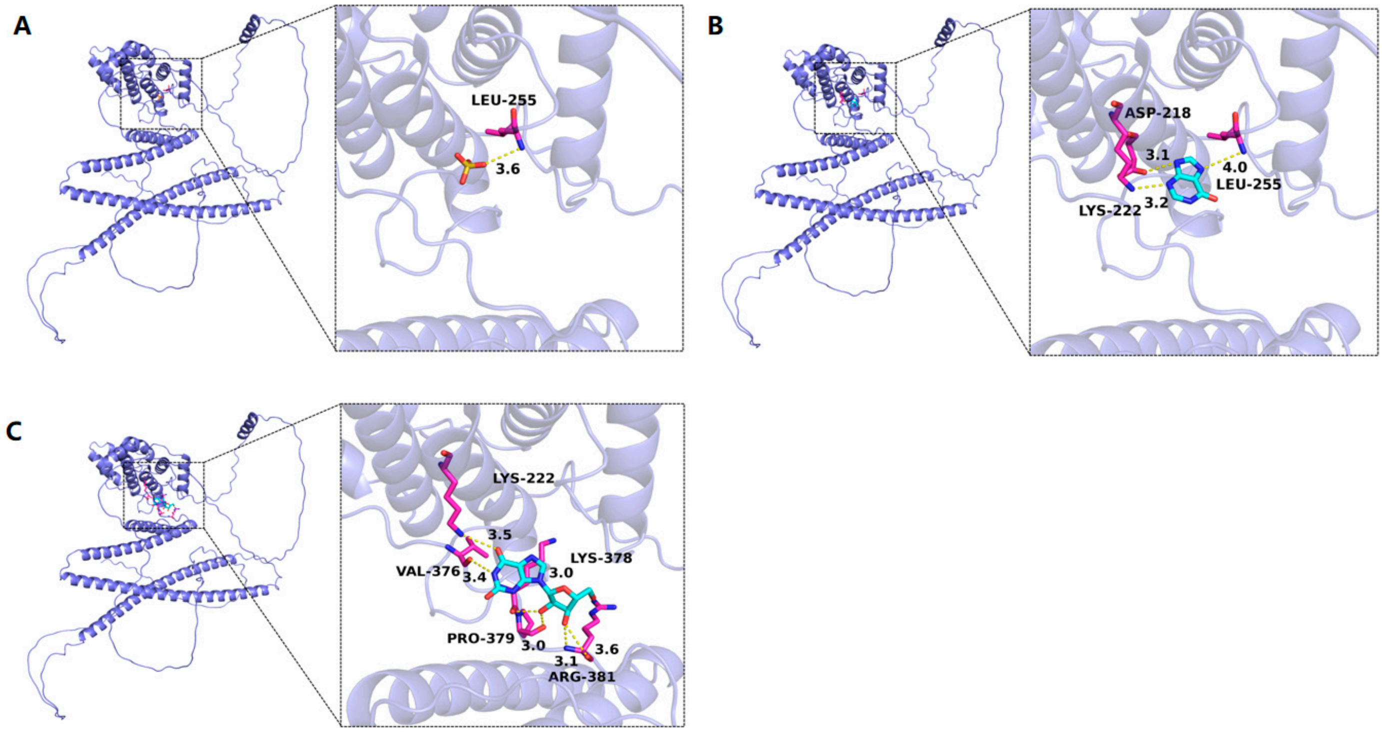

Supplementary Figure S7. Molecular docking analysis of JPT2 interactions with Sulfate, Hypoxanthine, and Xanthosine. The JPT2-Sulfate complex (A) shows only a single interaction via ASN41, corresponding to the weakest binding energy of –2.0 kcal/mol. The JPT2-Hypoxanthine complex (B) displays a moderate binding affinity (–4.1 kcal/mol), with key hydrogen bonds formed with ALA44, ASN46, and GLU52. Among the three, JPT2-Xanthosine (C) achieves the most favorable binding energy of –4.7 kcal/mol, characterized by a robust hydrogen-bond network involving GLY49, ASN46, PRO50, GLU52, and THR51.

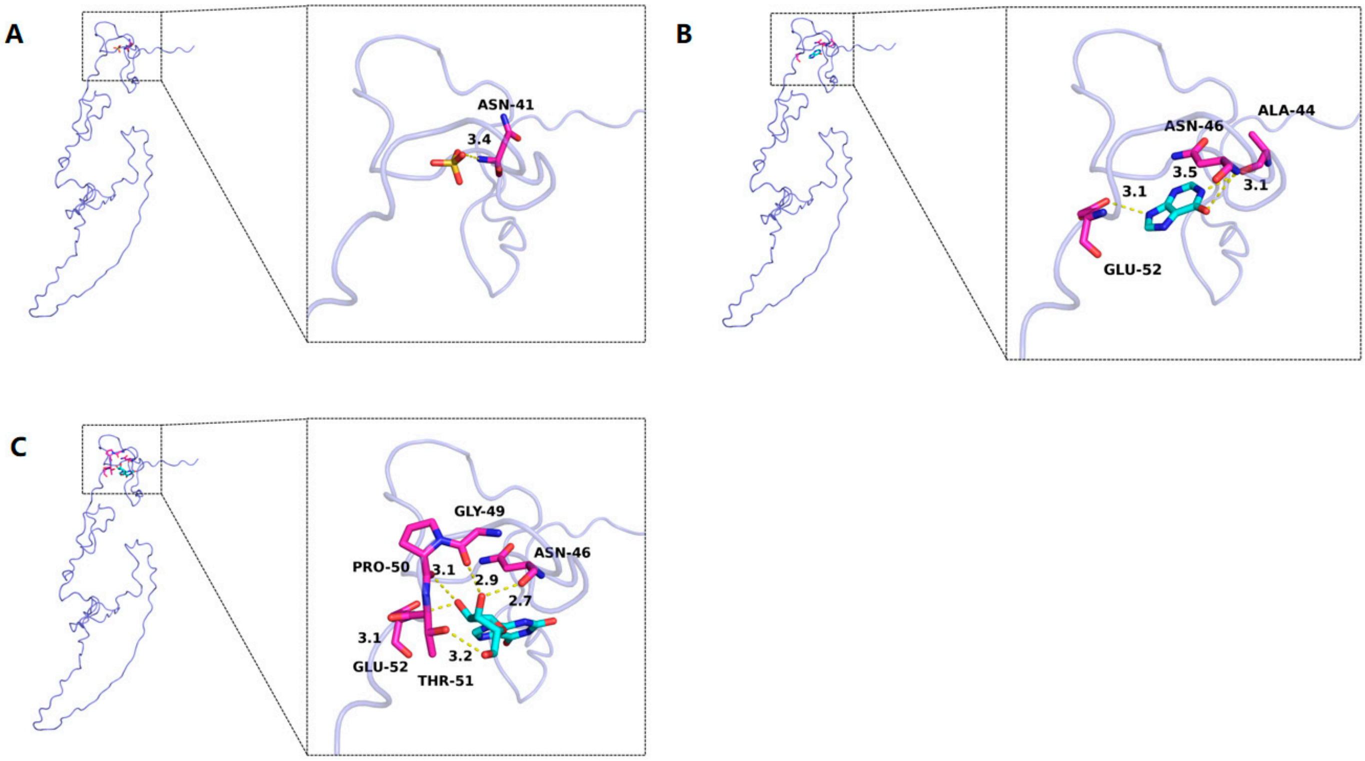

Supplementary Figure S8. Imaging features of all DIPG patients (enhanced T1WI).

(A) Patient 1; (B) Patient 2; (C) Patient 3; (D) Patient 4; (E) Patient 5; (F) Patient 6; (G) Patient 7; (H) Patient 8.

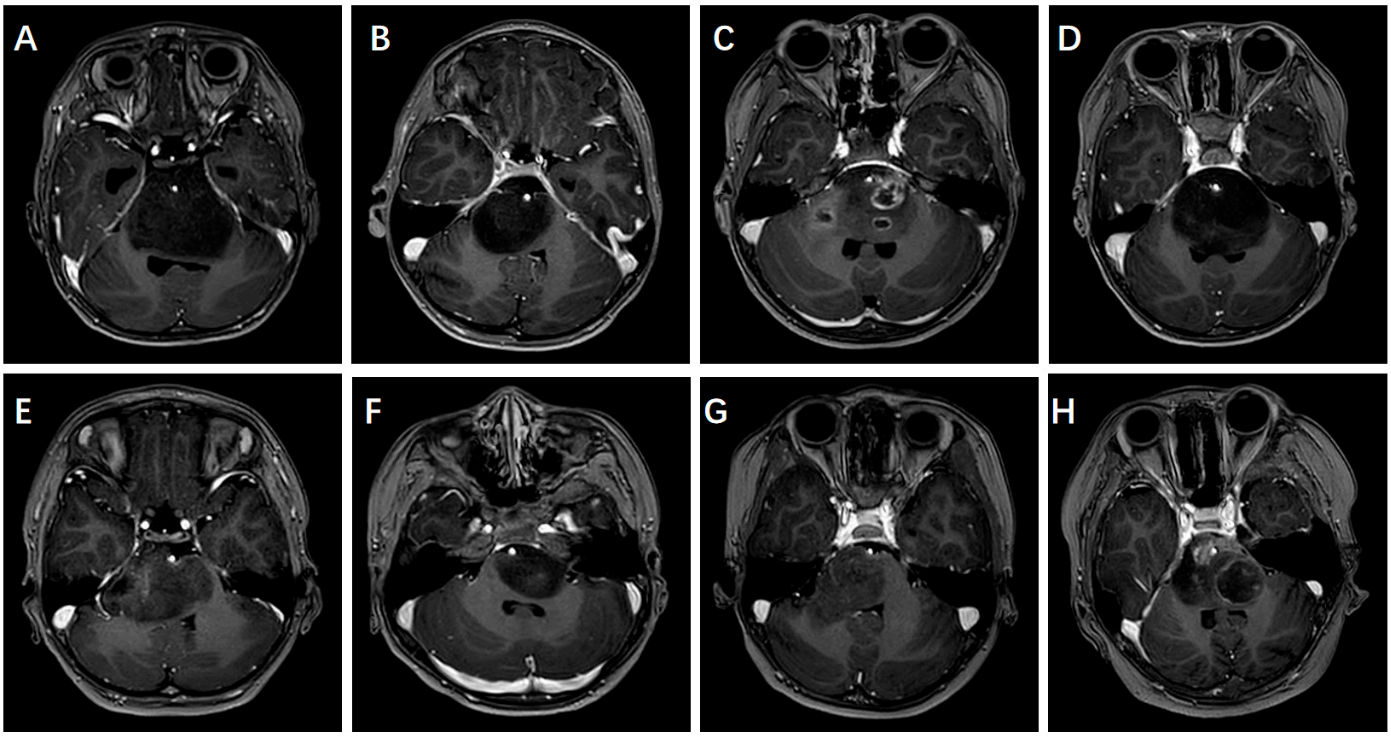

https://doi.org/10.3390/xxxxx
